# From Outpatient Consumption to Receiving Waters: A Retrospective Screening Assessment of Antibiotic Loads, Resistance Indicators, and Implications for the Recast Urban Wastewater Treatment Directive

**DOI:** 10.3390/antibiotics15100963

**Published:** 2026-09-29

**Authors:** Aneta Łuczkiewicz, Wojciech Artichowicz, Małgorzata Szopińska, Katarzyna Jankowska, Ola Svahn, Erland Björklund, Ewa Kotlarska, Nikol Szeszuła, Sylwia Fudala-Książek

**Affiliations:** 1 Faculty of Civil and Environmental Engineering, Gdansk University of Technology, Narutowicza 11/12, 80-233 Gdansk, Poland; wojciech.artichowicz@pg.edu.pl (W.A.); malgorzata.szopinska@pg.edu.pl (M.S.); katarzyna.jankowska@pg.edu.pl (K.J.); s189199@student.pg.edu.pl (N.S.); sylwia.fudala-ksiazek@pg.edu.pl (S.F.-K.); 2 Faculty of Natural Science, Kristianstad University, SE-291 39 Kristianstad, Sweden; ola.svahn@hkr.se; 3 University Executive Office, Malmo University, Neptuniplan 7, SE-211 18 Malmo, Sweden; erland.bjorklund@mau.se; 4 Genetics and Marine Biotechnology Department, Institute of Oceanology Polish Academy of Sciences, Powstańców Warszawy 55, 81-712 Sopot, Poland

**Keywords:** urban wastewater, antimicrobial agents, predicted incoming load, measured incoming load, receiving waters, environmental/antimicrobial risk assessment

## Abstract

**Background/Objectives**: Wastewater treatment plants (WWTPs) are recognized point sources of pharmaceutical residues and antimicrobial-resistant bacteria. This challenge is explicitly addressed in the 2024 recast of the Urban Wastewater Treatment Directive (UWWTD), which introduces risk-based obligations for micropollutant removal. **Methods**: Here, five antibiotics (azithromycin, clarithromycin, erythromycin, ciprofloxacin and sulfamethoxazole) were retrospectively (2017–2018) assessed in influent and effluent of four WWTPs and in their receiving waters (riverine and marine outfalls), including the Vistula Estuary. Measured incoming load (*MIL*) was compared with predicted incoming load (*PIL*), a cost-efficient proxy derived from outpatient consumption data. **Results:** The screening-level *PIL*–*MIL* consistency check showed closer agreement in catchments without major hospital inputs. In WWTPs, ciprofloxacin showed consistently high apparent aqueous-phase removal in both campaigns (>95%), sulfamethoxazole showed moderate removal (64–85%), while macrolides exhibited highly variable and sometimes apparent negative removal, yielding effluent concentrations up to 3989 ng L^−^^1^ (azithromycin) and 2866 ng L^−^^1^ (clarithromycin). Receiving waters generally contained < 10 ng L^−^^1^ of the investigated antibiotics, but a low-dilution-capacity river showed a clear WWTP influence. Presumptive cefotaxime-resistant *Escherichia coli*, used as proxies for emerging ESBL/AmpC-associated resistance, reached ~5% and 20% of CFU downstream of WWTP discharges. Environmental (ERA) and AMR-related (AMR-RA) risk quotients (*RQ*s) were mostly <0.1. However, *RQ*s for azithromycin approached or exceeded unity in Gdańsk and Puck Bay. Coastal recipients are more vulnerable despite high dilution, since marine predicted no-effect concentrations (*PNEC*s) for antimicrobials are lower than those for freshwater. **Conclusions**: The study supports the value of retrospective baseline data and site-specific screening frameworks for risk-based prioritization under the recast UWWTD.

## 1. Introduction

The global consumption of antibiotics has been steadily increasing over recent decades, largely driven by economic growth and improved equity in healthcare access [1]. According to Klein et al. [2], who analysed pharmaceutical sales data across 67 countries, between 2016 and 2023, national-level antibiotic consumption rose in upper-middle- and lower-middle-income countries, while in the European Union (EU) and other high-income countries it decreased or remained relatively stable during the same period [2]. In 2024, community consumption of systemic antibacterials (ATC J01) in the EU/EEA (EU Member States, Iceland and Norway) was estimated at 18.8 defined daily doses (DDD) per 1000 inhabitants per day (ECDC, 2024) [3], representing a 16% decrease compared with 2015 levels (ECDC, 2016) [3] (Table S1). This likely reflects both long-term antimicrobial stewardship efforts promoting prudent use and a transient COVID-19-related reduction (with a partial rebound), driven by fewer outpatient visits, increased remote work, and stricter infection prevention and control measures [2,3]. For instance, in Poland, community consumption of systemic antibacterials was 26.2 DDD per 1000 inhabitants per day in 2015, decreasing to 17.1 in 2020, and rising again to 21.2 in 2024 (ECDC, 2016; ECDC, 2025) [3] (Table S1). It is worth noting that, at the same time, hospital consumption remained at about 1.3–1.4 DDD per 1000 inhabitants per day (ECDC, 2016; ECDC, 2025) [3] (Table S1). This trend may be attributed to the essential role of antibiotics not only in treating infectious diseases but also in serving as prophylactic agents during cancer chemotherapy and major surgical procedures, thereby ensuring the success of these treatments and interventions [4].

However, the continued rise in the use of WHO ‘Reserve’ group antibiotics, which include broad-spectrum and last-line agents, in both community and hospital settings suggests a concerning shift towards treatments targeting multidrug-resistant organisms, further exacerbating the problem of antimicrobial resistance (AMR) (ECDC, 2024; Table S1) [3].

To address the growing threat of AMR within a One Health framework, Council Recommendation (2023/C 220/01) urges EU Member States to reduce total human consumption of antibiotics by 20% by 2030 compared with 2019 levels and to strengthen integrated surveillance systems for antimicrobial consumption and resistance monitoring [5]. Other legal initiatives have also been undertaken to tackle this issue. The recast Urban Wastewater Treatment Directive (UWWTD) [6], released in late 2024, incorporates the One Health approach and reflects the recognition that urban wastewater is a significant source of antimicrobial agents, their metabolites, as well as antimicrobial-resistant bacteria and resistance genes (UWWTD, 2024; and FAO, UNEP, WHO, WOAH, 2022) [6,7]. To further reduce emissions of pharmaceuticals and other micropollutants, quaternary treatment will become mandatory for all urban wastewater treatment plants (WWTPs) serving populations of 150,000 PE and above [6]. For agglomerations between 10,000 and 150,000 PE, this requirement applies in areas designated as sensitive to micropollutant pollution, unless a risk assessment confirms no significant threat to human health or the environment including risks related to AMR dissemination [6].

The presence of pharmaceuticals in freshwater ecosystems has already been documented (e.g., [8,9,10,11]); however, some countries, such as Poland, lack monitoring data on the environmental occurrence of compounds originating from WWTP discharges [8]. Nevertheless, such data are critical for establishing baseline conditions that support the effective implementation of the recast UWWTD, and are crucial for assessing how efforts to reduce pharmaceutical consumption are reflected in their occurrence in both WWTPs’ effluents and receiving waters.

In light of these concerns, this study retrospectively analysed data obtained in August 2017 and February 2018 sampling windows to quantify the occurrence, loads and concentrations of selected antibiotics—macrolides, fluoroquinolones and sulfonamides, in influent and effluent from municipal WWTPs and in their receiving waters, with a focus on Polish coastal zones of the Baltic Sea. The Baltic Sea is a semi-enclosed inland sea with high ecological value, slow water exchange and long residence times. These conditions favour the accumulation of contaminants, may threaten biodiversity and compromise water quality, especially in coastal waters [12].

In this study, the antimicrobials—erythromycin, azithromycin, clarithromycin, ciprofloxacin and sulfamethoxazole—were selected because they are widely used in human medicine and have been prioritised for EU-wide monitoring under EU water policy. These substances have appeared, although not always concurrently, in successive EU surface-water Watch Lists established under the Environmental Quality Standards Directive (European Commission, 2015, 2018, 2020, 2022) [13,14,15,16]. In addition, macrolide antibiotics are addressed in the European Commission proposal to revise pollutant lists and environmental quality standards under the Water Framework Directive, the Groundwater Directive and the Environmental Quality Standards Directive (European Parliament, 2025) [17].

Selected antimicrobials were analysed in the influent and effluent of four wastewater treatment plants (WWTPs). Measured incoming loads (*MIL*s) (bottom-up approach) were compared with predicted incoming loads (*PIL*s) derived from consumption data and compound-specific human excretion fractions (top-down perspective). *PIL* was expected to provide a resource-efficient basis for future assessments of pharmaceutical burdens on aquatic ecosystems. Additionally, this study assessed the environmental risks (ERA) associated with the presence of the investigated antimicrobials in WWTP receiving waters. Risk quotients (*RQ*s) were calculated by comparing measured environmental concentrations (*MEC*s) with predicted no-effect concentrations (*PNEC*s) (NORMAN Network, 2024) [18]. The potential for antimicrobial-resistance selection in the environment (AMR-RA) was also evaluated by comparing *MEC*s with predicted no-effect concentrations for resistance (*PNEC*_R_). Moreover, faecal contamination was assessed by enumerating faecal coliforms (FC) and *Escherichia coli*, including isolates presumptively resistant to cefotaxime (CTX-R). This approach provides a culture-based screening indicator potentially relevant ESBL/AmpC-associated resistance among *Enterobacterales* at the human–environment interface.

## 2. Materials and Methods

### 2.1. Study Area

**Figure 1:**
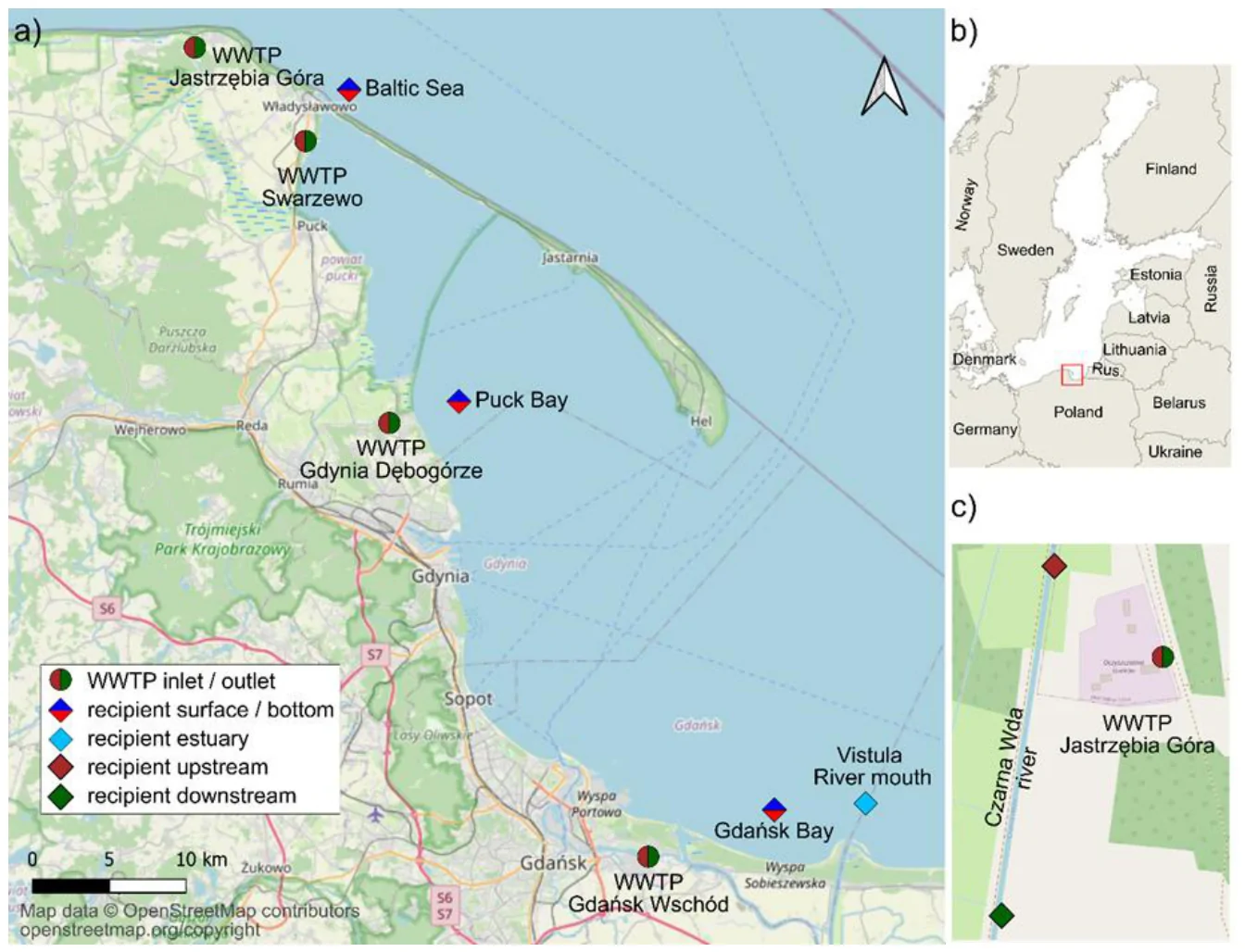
Location of the study area and sampling sites; (**a**) locations of the four wastewater treatment plants (WWTPs), where influent and effluent samples were collected, and the marine and estuarine sampling sites along the Polish Baltic coast; (**b**) location of the study area within the Baltic Sea region (red rectangle); (**c**) detailed view of the Czarna Wda River, showing the Jastrzębia Góra WWTP and the river sampling sites upstream and downstream of its effluent discharge Ref. [19].

The study area comprises four municipal WWTPs located in the Pomeranian Voivodeship (Figure 1). Three of them—Gdańsk-Wschód WWTP (GW-WWTP), Gdynia-Dębogórze WWTP (GD-WWTP) and Swarzewo WWTP (SW-WWTP)—are the largest in the region and discharge via submarine collectors into the coastal waters of Gdańsk Bay, its inner part Puck Bay, and the Baltic Sea, respectively. The outfalls of GW-WWTP and GD-WWTP extend approximately 2.3–2.5 km offshore, while the SW-WWTP outfall is located about 1.4 km from the shoreline (Table S2a). Treated wastewater is conveyed offshore under pressure via pumping stations, with the outfall systems equipped with diffuser sections intended to support dilution and dispersion in the receiving waters.

The fourth plant, Jastrzębia Góra WWTP (JG-WWTP), discharges into the Czarna Wda (CW) River, approximately 4 km upstream of its mouth into the Baltic Sea. All four WWTPs are situated in the coastal zone and are influenced by tourism. The highest wastewater flows are observed during the summer season, particularly at SW-WWTP and JG-WWTP. Characteristics of the studied WWTPs, including information on discharge permits, are presented in Table S2a,b.

In addition, the Vistula River mouth (VR) was also included as a sampling site. The Vistula is the longest river in Poland (1047 km), with a catchment area of 194,424 km^2^, draining a large part of the country. It flows directly into the Gulf of Gdańsk, with an average annual discharge of 1054 m^3^/s. The resulting river plume can extend between 9 and 27 km from the river mouth, depending primarily on weather conditions (Figure 1).

### 2.2. Sampling

Two sampling campaigns were conducted as part of this research: the first from August 23 to 30, 2017 (summer period), and the second from February 27 to 28, 2018 (winter period). A total of 16 samples were collected from the WWTPs using 24 h continuous flow-proportional automatic samplers—from both the influent (after mechanical treatment) and the effluent (after the final clarifier). The influent, effluent and receiving-water sampling was synchronized with the hydraulic retention time (approximately from half to one day) (Figure 1). Coastal zones receiving effluents from these facilities were sampled directly close to the diffusers mounted at a depth of about 8 m (bottom water samples, BOT) and at the surface above submarine collectors (surface water samples, SUR). Additionally, surface water from the Vistula River mouth (VR-M) was collected. In the case of JG-WWTP, which discharges into the Czarna Wda River, additional samples were taken upstream (CW-UP) and downstream (CW-DOWN), approximately 400 m from the discharge point (Figure 1). All samples from the WWTP receiving waters and the Vistula River were collected using manual grab sampling using a Nansen bottle, which allows depth-specific collection of discrete water samples. Altogether 17 samples were collected, because marine near-bottom water samples from the SW-WWTP submarine outfall area were not collected in February due to adverse weather conditions.

The samples were collected without headspace into airtight, chemically clean bottles. The cleanliness of the sampling procedure was verified by the daily collection of field blanks. Prior to sampling, bottles were pre-cleaned by soaking in Milli-Q deionised water for one week, followed by several rinse-and-drain cycles. For microbiological analyses, samples were collected into sterile glass bottles. All samples were transported to the laboratory at 4 °C. Physicochemical and microbiological analyses were performed on the day of collection, while samples intended for antimicrobial analysis were stored at −20 °C and analysed within one month.

### 2.3. Analysis of Standard Parameters

Basic chemical parameters of the inlet and outlet wastewater samples, as well as receiving waters (fresh or marine water samples) were characterized by: chemical oxygen demand (COD), total nitrogen (TN), inorganic N compounds (N-NH_4+_, N-NO_3−_, N-NO_2−_), total phosphorus (TP) and orthophosphate (P-PO_4_^3−^) using cuvette spectrophotometric tests together with DR 3900 spectrophotometer (HACH Lange, GmbH, Düsseldorf, Germany). Chloride (Cl^−^) and sulphate (SO_4_^2−^) ion concentrations were measured by ion chromatography (IC) using: a DIONEX 3000 chromatograph (DIONEX, Sunnyvale, CA, USA) (column: Ion Pac^®^AS22 [2 × 250 mm]; suppressor: ASRS-300, 2 mm; mobile phase: 4.5 mM CO_3_^2−^, 1.4 mM HCO_3_^−^; flow rate: 0.38 mL/min; conductivity detection). Reference standards for IC were purchased from Supelco (Bellefonte, PA, USA): (1) traceable to SRM from NIST NaCl in H_2_O 1000 mg L^−^^1^ Cl^−^ Certipur^®^ reference materials (Merck KGaA, Darmstadt, Germany) and (2) traceable to SRM from NIST Na_2_SO_4_ in H_2_O 1000 mg L^−^^1^ SO_4_^2−^ Certipur^®^. Moreover, pH, electrochemical conductivity and oxidation-reduction potential were determined by a portable multi-parameter meter, the HL-HQ40d multi (HACH Lange, GmbH, Düsseldorf, Germany), and 5-day biochemical oxygen demand (BOD_5_) by manometric respirometric BOD OxiTop^®^ method. Total suspended solids (TSS), volatile suspended solids (VSSs) and mineral suspended solids (MSS) were measured by the gravimetric method. All chemical and physical parameters are reported in Supplementary Table S2c,d. As samples were collected in August 2017 and February 2018, the average monthly wastewater flows reported by the selected WWTPs for these months (Table S2e) were used to calculate the loads of measured parameters discharged from the WWTPs to the receiving waters.

### 2.4. Analysis of Antibiotics

The selected antibiotics (macrolides: azithromycin, clarithromycin and erythromycin; fluoroquinolone: ciprofloxacin, and sulfonamide: sulfamethoxazole) were analysed by ultra-high performance liquid chromatography electrospray ionization tandem mass spectrometry (UPLC-ESI-MS/MS) after solid-phase extraction (SPE) [20,21]. For UPLC-ESI-MS/MS analysis, ultra-pure water (18.2 MΩ·cm) was obtained from an OPTIMA water purification system (Elga Ltd., High Wycombe, Buckinghamshire, UK). Reference standards were purchased from Sigma-Aldrich Sweden AB (Stockholm, Sweden). Mobile phase chemicals including: acetonitrile and methanol, were both HPLC-grade and purchased from Fisher Scientific (Gothenburg, Sweden) or Sigma-Aldrich (Steinheim, Germany). Additives to mobile phase: formic acid, ammonium hydroxide solution (25% sol.), ammonium hydrogen carbonate, disodium ethylenediaminetetraacetate (Na_2_EDTA), ascorbic acid, and ammonium hydroxide were purchased from Sigma-Aldrich (Steinheim, Germany). Clean pressurized air was produced by an Atlas Copco SF2 oil-free air system (Atlas CopcoAirpower n.v. B, Wilrijk, Belgium).

Inlet and outlet wastewater samples (50 g each) were applied on SPE cartridges (Oasis HLB 200 mg, 6 mL) with the finely ground sand pre-filtration (2 g) using traditional SPE set-up with the negative pressure application. Freshwater and marine water samples were prepared using high flow-rate sample loading (500 g each) with the application of positive pressure and finely ground sand (2 g) as an SPE-column in-line filter [21]. All samples were applied to the SPE columns unfiltered. Wastewater samples—50 g were weighed and 50 μL formic acid (10%) and 50 μL EDTA (sat. sol.) were added together with 30 μL of IS mixture. A 500 g water sample was weighed and 100 μL formic acid (10%), and 100 μL EDTA (sat. sol.) was added together with 30 μL of IS mixture. Next, samples were dried under a stream of air (hospital grade) for 20 min. Elution was performed using 6 mL of methanol. Next, samples were evaporated under a gentle stream of compressed air (appx. 22 min). As a final step, samples were reconstituted in the 100 μL of methanol, 885 μL of deionised water, 10 μL EDTA (sat. sol.) and 5 μL of an instrumental internal standard and injected to UPLC-ESI-MS/MS in 1 μL volume. Method quantification limits (MQLs) were determined separately for wastewater and receiving waters. In wastewater, MQLs were 1.1 ng L^−1^ for azithromycin and clarithromycin, 32 ng L^−1^ for ciprofloxacin, 1.3 ng L^−^^1^ for sulfamethoxazole, and 0.5 ng L^−^^1^ for erythromycin. In receiving waters, the corresponding MQLs were 0.1, 0.2, 0.9, and 0.5 ng L^−^^1^, respectively. For aqueous samples, the matrix effect (matrix-rich pond water), expressed as relative recovery, was as follows: azithromycin (Ciprofloxacin-D_8_): 87.1–382.4%; clarithromycin (Clarithromycin-D_3_): 78.3–105.8%; erythromycin (Clarithromycin-D_3_): 100.6–112.7%; ciprofloxacin (Ciprofloxacin-D_8_): 26.6–108.4%; and sulfamethoxazole (Sulfamethoxazole-^13^C_6_): 116.7–119.3%. The corresponding absolute recoveries of the internal standards were 21.4–37.9% for Ciprofloxacin-D_8_, 54.7–70.9% for Clarithromycin-D_3_, and 94.4–106.3% for Sulfamethoxazole-^13^C_6_. A detailed description of the method validation parameters and chromatographic conditions is provided in Svahn and Björklund (2016) and Svahn and Björklund (2019) [20,21].

### 2.5. Microbiological Analyses

Thermotolerant coliforms (hereafter, faecal coliforms, FC) and *Escherichia coli* (*E. coli*) were enumerated by membrane filtration using m-FC agar supplemented with rosolic acid (Merck, Germany), according to ISO 9308-1:2014 [22]. The filtration volume was adjusted according to the sample type (raw wastewater, treated wastewater, river and seawater) to ensure that colony numbers fell within the predefined countable range; 10–200 CFU per plate was considered optimal. Wastewater samples were serially diluted and appropriate aliquots were filtered through sterile nitrocellulose membrane filters (0.45 µm pore size). For both raw and treated wastewater, serial dilutions ranging from 10^0^ to 10^−6^ were prepared and filtered. In addition, undiluted samples were filtered at a volume of 1 mL for raw wastewater and 10 mL for treated wastewater. Seawater and river water samples were filtered at volumes ranging from 250 mL to 2 L, depending on the expected microbial concentration. Membranes from each dilution/sample were placed on m-FC plates in triplicate (with the three plates representing technical replicates) and incubated at 44.5 °C for 22–24 h. Blue colonies were recorded as FC. FC colonies were subsequently subjected to confirmatory testing for *E. coli* using established criteria: oxidase negativity, citrate negativity, gas production in EC broth, and indole positivity. Counts were normalised to the filtered volume and reported as colony-forming units (CFU) per mL of sample.

The measured *E. coli* concentrations in marine and riverine samples were compared only as contextual, single sample values with the numerical benchmark values provided in Directive 2006/7/EC. They should not be interpreted as a formal bathing-water classification, because such classification requires percentile-based assessment of an adequate bathing-water quality dataset and includes both *E. coli* and intestinal enterococci. Therefore, the results are reported only as location- and time-specific microbiological observations.

In parallel, cefotaxime-resistant FC and *E. coli* (CTX-R FC and CTX-R *E. coli*) were enumerated using m-FC agar supplemented with cefotaxime sodium salt at a final concentration of 2 µg mL^−1^. This screening concentration corresponds to the EUCAST cefotaxime MIC breakpoints for *Enterobacterales* [23]. Blue colonies growing on cefotaxime-supplemented plates were counted as presumptive CTX-R FC and were subjected to the same confirmatory tests; isolates fulfilling all criteria were classified as presumptive CTX-R *E. coli*.

### 2.6. Outpatient Consumption Data for Tested Antimicrobial Agents

In this study, outpatient antibiotic consumption data were obtained from the National Health Fund (NFZ) under a special data request. The dataset covers nationwide community-pharmacy dispensations of reimbursed prescription medicines and excludes inpatient hospital use; in Poland, antibiotics are not available over the counter. For each product, the dataset included the EAN code, detailed product information (amount of active substance, number of units per pack or volume in millilitres), and monthly counts of dispensed packages. Focusing on the Pomeranian Voivodeship, we calculated the total monthly consumption for each antibiotic (with particular attention to August 2017 and February 2018, when environmental samplings were conducted).

### 2.7. Calculation of Predicted Incoming Loads (PIL) and Measured Incoming Loads (MIL)

To link community antibiotic use with the mass of pharmaceuticals entering the studied WWTPs, two complementary approaches were applied:

(i) A top-down, consumption-based approach used to estimate *PIL*s: for each antibiotic and each study month (August 2017 and February 2018), *PIL* was calculated from outpatient consumption data (Section 2.6) and the size of the population connected to a given WWTP (Table S2a), assuming that the excreted fraction (Table S3) of the parent compound enters the sewer network; the following equation was used:
  (1) $$PIL=C\cdot f_{excr}\cdot N$$
  where:
  *PIL*—predicted incoming load (kg∙month^−1^),
  *C*—antimicrobial agent consumption in the Pomeranian Voivodeship (kg∙inhabitant^−1^∙month^−1^),
  *f_excr_*—excretion fraction of antimicrobial agent excreted as a parent compound (-),
  *N*—number of inhabitants connected to the WWTP (-).
(ii) A bottom-up, monitoring-based approach, used to derive *MIL*s: for each study month (August 2017 and February 2018), influent concentrations of the antimicrobials and corresponding flows were used to calculate mass load in the WWTP influent; the following equation was used:
  (2) $$MIL=c_{in}\cdot Q_{in}\;\cdot 10^{-9}$$
  where:
  *MIL*—measured incoming load (kg∙month^−1^),
  *c_in_*—measured concentration of antimicrobial agent (ng L^−1^),
  *Q_in_*—monthly influent volume (m^3^ month^−1^),
  $10^{-9}$—units conversion factor (resulting from ng to kg and L to m^3^).

The resulting value should not be interpreted as a directly measured monthly load but rather as an estimate of the monthly load based on a 24 h measured concentration.

### 2.8. Environmental Risk Assessment (ERA) and Antimicrobial Risk Assessment (AMR-RA)

This study also evaluated the risks associated with the presence of antimicrobial agents in aquatic environments receiving treated wastewater effluents. In addition to conventional environmental risk assessment (ERA), the potential for the selection of antimicrobial resistance in the environment (AMR-RA) was also addressed due to the use of antimicrobial compounds. According to the guidelines from the European Medicines Agency (EMA) [24], ERA is typically based on compound-specific threshold values such as the predicted no-effect concentration (*PNEC*). For AMR-RA, a similar approach is applied using predicted no-effect concentrations for resistance selection (*PNEC*_R_). Both *PNEC* and *PNEC*_R_ values are commonly derived from experimentally determined no observed effect concentrations (NOEC and NOEC_R_, respectively), divided by an appropriate assessment factor (AF), which typically ranges from 10 to 1000. However, it should be noted that a standardized experimental methodology for the routine determination of *PNEC*_R_ is still lacking. Table 1 presents the *PNEC* and *PNEC*_R_ values used in this study. The lowest *PNEC* values for fresh and marine waters were retrieved from the NORMAN database [18], a collaborative European platform that compiles monitoring and ecotoxicological data for emerging substances and serves as a key resource for environmental risk assessment and regulatory purposes. *PNEC*_R_ values were compiled using the approaches suggested by Bengtsson-Palme & Larsson [25]—extrapolation of MIC dataset for individual clinical strains and, as suggested by Murray et al. [26], wastewater influent growth was tested using the SELECT statistic experimental system.

In both ERA and AMR-RA, risk was evaluated using the risk quotient (*RQ*) method, in which *RQ* can be calculated as the ratio of measured (*MEC*) or predicted environmental concentrations (PEC) of pharmaceuticals to the respective threshold (*PNEC* or *PNEC*_R_). In this study, *MEC* was used (see Equations (3) and (4)). An *RQ*/*RQ*_R_ value greater than 1 indicates a potential environmental or resistance-related risk.

(3) $$RQ=\frac{MEC}{PNEC}$$

(4) $${RQ}_{R}=\frac{MEC}{{PNEC}_{R}}$$

where:

*MEC*—measured environmental concentration of tested antimicrobial agents;
*PNEC*—predicted no-effect concentration in the relevant compartment;
*PNEC*_R_—predicted no-effect concentration for resistance.

## 3. Results and Discussion

The current interpretation of WWTP performance is framed by the transition from the Council Directive 91/271/EEC on urban wastewater treatment (UWWTD 1991) [27], focused mainly on conventional pollutant removal which did not fully address other emerging pressures [28], to the 2024 recast UWWTD [6], which strengthens nutrient-management requirements and introduces a broader focus on micropollutants and wastewater-based One Health surveillance. This context is relevant for interpreting both the classical nutrient-removal results and the screening-level assessment of antimicrobial loads and receiving-water risks presented below.

This retrospective two-campaign screening study examined four coastal WWTPs in northern Poland and provides baseline data on nutrient and antimicrobial loads and associated pressures on Baltic Sea receiving waters, relevant to future risk-based implementation of the recast UWWTD. During the August 2017 and February 2018 sampling windows, WWTP influent and effluent were analysed together with samples collected at the corresponding receiving-water sites (Figure 1). The campaigns were designed to maximise comparability between WWTP effluent and receiving-water data. Influent and effluent sampling at the WWTPs was scheduled to account for hydraulic retention time, whereas receiving water samples were collected during the corresponding effluent-sampling window. It was of particular importance for marine samples at offshore sites located approximately 1.4–2.5 km from the shoreline, in the vicinity of the wastewater discharge zones. Such WWTP–marine receiving-water datasets are scarce, particularly in Poland, and provide valuable screening-level information.

The Gulf of Gdańsk, including Puck Bay in its inner western part, is subject to multiple anthropogenic pressures, including municipal wastewater discharges, local riverine inputs, inflow from the Vistula River, maritime transport, and tourism and recreational activities. Therefore, the Vistula River mouth was included in the study to provide broader context for the coastal receiving-water environment.

### 3.1. Physicochemical Characteristics in WWTP Inflows, Outflows, and Receiving Waters

Based on the obtained results (Table S2c,d), the basic WWTP parameters of influent quality: TP (7.9–19.4 mg P L^−1^), TN (70.4–146.2 mg N L^−1^), BOD (360–945 mg O_2_ L^−1^), COD (765–1375 mg O_2_ L^−1^) and TSS (340–624 mg L^−1^) were within typical range of municipal wastewater [29,30]. The treatment performance of studied WWTPs resulted in effluent that complied with the current discharge limits (see Table S2b for comparison). However, GD-WWTP, SW-WWTP, and JG-WWTP exceeded the stricter TP thresholds proposed under the recast UWWTD, and JG-WWTP also exceeded the corresponding threshold for TN (Figure S1 and comments in Supplementary Materials).

In the studied months (August 2017 and February 2018) the loads of organic matter and nutrients discharged into the receiving waters were calculated (Table S2e). In August 2017, the two largest WWTPs, GW-WWTP (840,200 PE) and GD-WWTP (440,000 PE), together released more than 31 Mg of total nitrogen (TN) and 2.5 Mg of total phosphorus (TP) into the Gulf of Gdańsk and its inner basin, Puck Bay, with more than 65% of these nutrient loads present in mineral forms, primarily as nitrate and phosphate.

In the case of GW-WWTP and GD-WWTP, treated effluents are discharged via marine outfalls situated more than 2 km offshore in the Gulf of Gdańsk/Puck Bay receiving-water system, whereas SW-WWTP discharges treated effluent to the open Baltic Sea near Władysławowo through a marine outfall located approximately 1.4 km from the shoreline. For these outfalls, near-bottom water samples collected directly above the diffuser zones showed concentrations comparable to those measured in surface waters (Figure S1). This pattern is consistent with initial dilution and/or mixing under the sampled conditions and is compatible with the intended function of the diffuser systems to enhance effluent dispersion. A definitive assessment of hydrodynamic mixing efficiency and long-term receiving-water impact would require dedicated hydrodynamic measurements, repeated sampling and/or dispersion modelling.

In contrast, in the Czarna Wda River, higher concentrations were observed downstream than upstream of the JG-WWTP discharge point, particularly in August 2017. This pattern is consistent with local wastewater influence and may reflect reduced dilution capacity under the sampled summer conditions.

The Vistula River was also included in the study, as it is a major river discharging into the Baltic Sea. According to the obtained results, TN and TP concentrations in the river estuary did not exceed 1.0 mg N L^−1^ and 0.1 mg P L^−1^, respectively, with slightly lower values recorded during the winter compared to the summer season (Figure S1). It is noteworthy that over the past three decades, a significant and statistically meaningful reduction in nutrient loads discharged into the Baltic Sea from Polish catchments has been observed. In the early 1990s, nutrient concentrations in the Vistula River were approximately five times higher [31], and this decrease is linked primarily to regulatory frameworks mandating the implementation of wastewater collection and tertiary treatment systems. Nonetheless, there is a need to enhance optimization of wastewater treatment technologies to meet the forthcoming 2045 standards. This may require addressing several operational challenges and improving control of WWTP influent-related factors, including local industrial discharges [32].

### 3.2. Microbiological Characteristics in WWTP Inflows, Outflows, and Receiving Waters

Currently, there are no specific EU requirements regulating the microbial quality of treated wastewater discharged into receiving water bodies. However, the recast UWWTD [6] introduces provisions for monitoring, e.g., AMR in wastewater, aiming to strengthen the One Health approach and to enhance scientific knowledge for future evidence-based interventions. Anticipating these regulatory developments, this study conducted microbiological analyses to assess the faecal pollution indicators (FC and *E. coli*) in WWTP influent, effluent, and receiving waters. In addition, in line with WHO [33], the occurrence of CTX-R strains within the faecal indicators was tested, to provide insights into AMR prevalence and dissemination of β-lactam resistance among FC and *E. coli* in tested niches.

**Figure 2:**
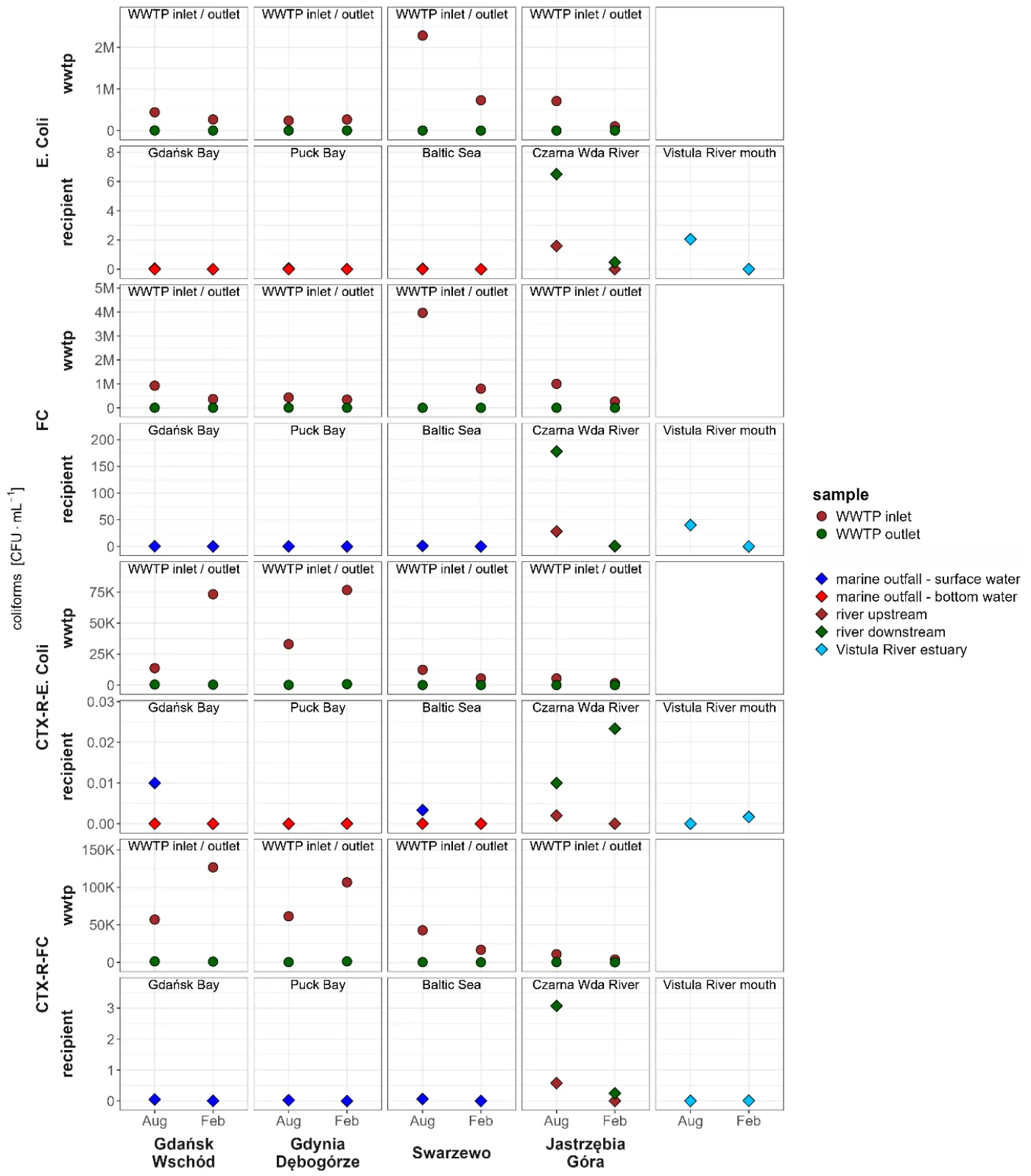
Occurrence of faecal coliforms (FC) and *E. coli*, including presumptive cefotaxime-resistant strains (CTX-R FC and CTX-R *E. coli*), in the inlets and outlets of tested WWTPs in: Gdańsk Wschód, Gdynia Dębogórze, Swarzewo, and Jastrzębia Góra, and in their receiving waters: surface and bottom waters in the marine outfalls areas in Gdańsk Bay, Puck Bay, Baltic Sea as well as from downstream and upstream of the discharge point in the Czarna Wda River; additionally the results are presented for Vistula River mouth (see Figure 1 for details).

According to the results (Figure 2), the numbers of faecal coliforms (FC) and *E. coli* in raw wastewater varied between the summer and winter sampling periods, ranging from approximately 2.6 × 10^5^ to 4.0 × 10^6^ CFU/mL for FC and from 1.0 × 10^5^ to 2.3 × 10^6^ CFU/mL for *E. coli*, which is consistent with values typically reported for municipal wastewater [34,35]. Wastewater treatment processes reduced *E. coli* levels by three to four orders of magnitude, with the highest removal efficiency observed at JG-WWTP, where UV disinfection is applied. Such generally high removal efficiency (>99%) is usually reported for faecal indicators and other bacteria in conventional WWTP based on activated sludge [35]. However, due to their high initial concentrations in raw sewage, the loads of FC and *E. coli* in treated wastewater still result in the continuous release of substantial numbers of these bacteria into receiving waters. For instance, GW-WWTP and GD-WWTP together discharged more than 10^16^ CFU of FC and *E. coli* into the Gulf of Gdańsk and Puck Bay, with presumptive CTX-R CFU accounting for up to 35% of CFU recovered from treated effluents (Table S2e). One higher value was observed for CTX-R FC/FC at GW-WWTP in February 2018 (57.1%). In general, this proportion is in agreement with findings from the global study by Marano et al. [35], although it falls within the upper range of the values reported.

Within the WHO Global Antimicrobial Resistance and Use Surveillance System (GLASS), *E coli* resistant to third-generation cephalosporins (e.g., CTX-R *E. coli*) is used as a key surveillance indicator and proxy for ESBL- and/or AmpC-associated resistance phenotypes among *Enterobacterales* [33]. In clinical settings, this resistance phenotype is often associated with increased use of antibiotics from the WHO ‘Watch’ group and, in more severe infections, may necessitate treatment with agents from the ‘Reserve’ group. The occurrence of CTX-R *E. coli* in wastewater and receiving waters may indirectly reflect environmental pressures and, in this context, the AMR component provides complementary One Health information and a retrospective baseline relevant to emerging EU requirements for AMR monitoring in urban wastewater and receivers.

As mentioned above, the sanitary quality of treated wastewater has not been regulated by previous and recast UWWTD [6,27]; however, the quality of receiving waters, particularly designated bathing areas, is monitored and regulated under Directive 2006/7/EC [36]. The measured *E. coli* concentrations were compared only as contextual single-sample values with the numerical benchmark values provided in Directive 2006/7/EC. They should not be interpreted as formal bathing-water classification, because such classification requires percentile-based assessment of an adequate bathing-water quality dataset and includes both *E. coli* and intestinal enterococci. Therefore, the results are reported only as location- and time-specific microbiological observations. To achieve an ‘excellent’ or ‘sufficient’ classification in coastal waters, *E. coli* concentrations must be limited to 250 and 500 CFU/100 mL, respectively, based on a 95th percentile evaluation. At the tested discharge points of GW-WWTP, GD-WWTP, and SW-WWTP, *E. coli* numbers were either below the detection limit or did not exceed 10 CFU/ mL, suggesting that the submarine outfall systems may minimise impacts on coastal areas under the sampled conditions (Figure 2). In the case of JG-WWTP, although this facility applies UV disinfection, the *E. coli* number in the Czarna Wda River (CW-R) still exceeded the threshold for “excellent” inland water classification during the summer season, reaching 650 CFU/100 mL downstream of the discharge point. Nonetheless, the water quality remained within the “sufficient” classification limit (900 CFU/100 mL). In addition, analysis of presumptive CTX-R CFU downstream of the outfall revealed an elevated contribution in winter samples (February 2018), accounting for approximately 5% of *E. coli* CFU and up to 20% of FC CFU. These observations highlight the need to investigate not only antimicrobial-resistant strains but also the dissemination of AMR indicators and transport of pharmaceutical residues in receiving waters with low dispersion potential.

### 3.3. Influent and Effluent Concentrations of the Target Pharmaceuticals in WWTPs

**Figure 3:**
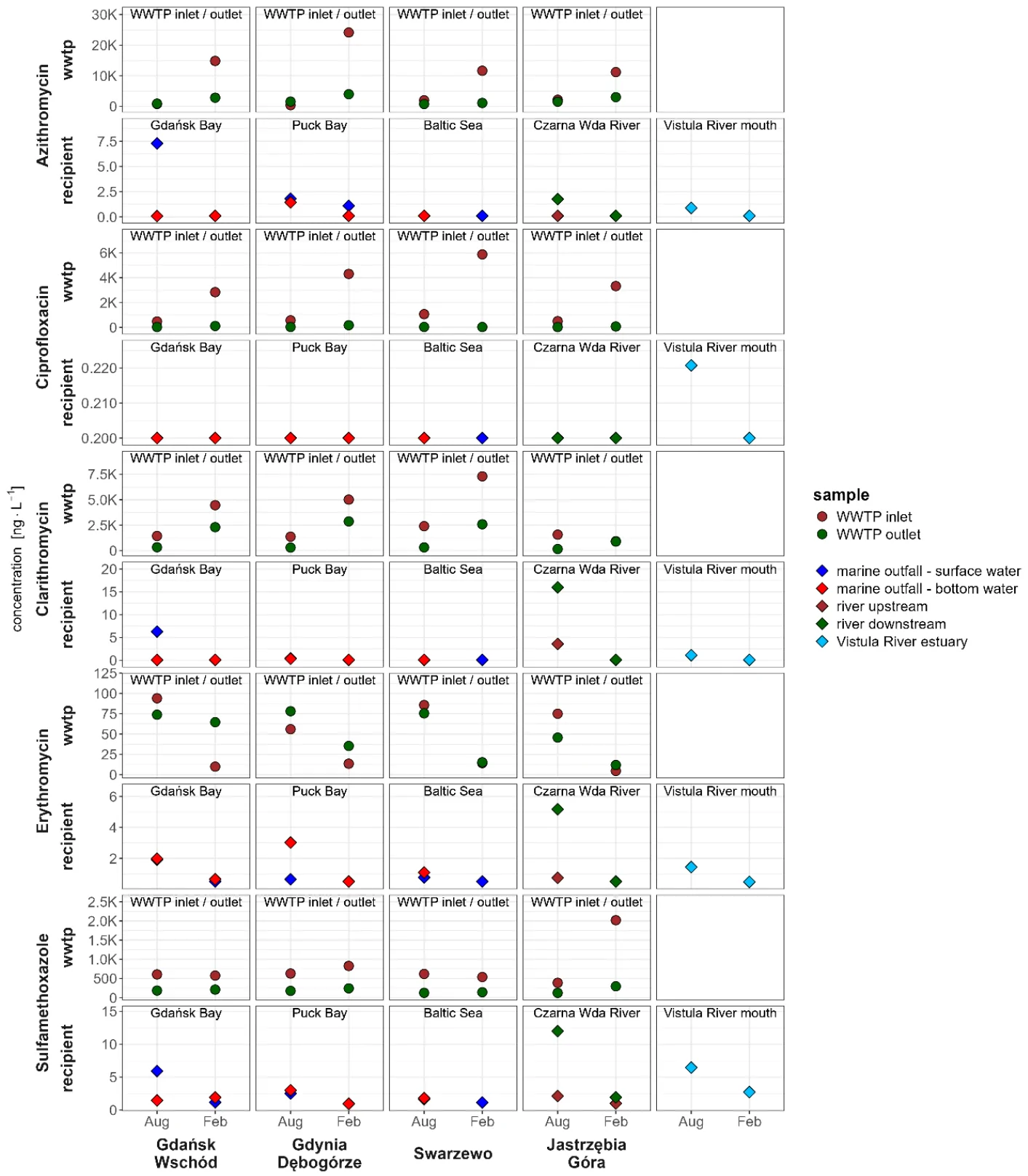
Concentrations of erythromycin, azithromycin, clarithromycin, ciprofloxacin and sulfamethoxazole in influent and effluent of WWTPs in Gdańsk-Wschód, Gdynia-Dębogórze, Swarzewo and Jastrzębia Góra, and in their receiving waters: surface and bottom waters in the marine outfall areas of Gdańsk Bay, Puck Bay and the Baltic Sea, as well as upstream and downstream of the discharge point in the Czarna Wda River; additionally the results are presented for Vistula River mouth (see Figure 1 for details).

In general, across the influents of all WWTPs included in the study (Figure 1), the highest concentrations of antibiotics (macrolides: erythromycin, azithromycin and clarithromycin; fluoroquinolone: ciprofloxacin; and sulfonamide: sulfamethoxazole) were observed during the February 2018 sampling window (Figure 3). Among the target compounds, azithromycin showed the most pronounced occurrence in influent wastewater, with concentrations ranging from 403.5 to 6036.2 ng L^−1^ across the investigated WWTPs, the maximum being observed at GD-WWTP. Clarithromycin was the next most abundant compound, with concentrations ranging from 922.5 to 7294.1 ng L^−1^.

This pattern is consistent with antimicrobial consumption data from the EU/EEA, which show that, at the community level, macrolides (classified together with lincosamides and streptogramins as ATC group J01F) were the second most frequently used group of antibacterials after β-lactams (ATC groups J01C/J01D) [3]. Among macrolides, the predominance of azithromycin and clarithromycin in raw wastewater samples likely reflects their more frequent use in outpatient and other community-care settings compared with erythromycin (see also Section 3.5 for comparison), due to more favourable pharmacokinetic properties, broader antimicrobial spectra and better tolerability. In this study, erythromycin was detected at the lowest concentrations among the tested antimicrobials, ranging from 4.6 to 94 ng L^−1^ (Figure 3). Erythromycin was followed by sulfamethoxazole (387.5–2019.8 ng L^−1^) (most commonly used in combination with trimethoprim, as co-trimoxazole) and ciprofloxacin (474.7–5873.2 ng L^−1^), both important agents in the treatment of urinary tract infections and selected respiratory and gastrointestinal infections.

Given the influent concentrations of antimicrobial agents described above, their removal efficiencies in the studied WWTPs varied markedly between compounds. Irrespective of the sampling window, the highest rate, close to or exceeding 95%, were observed for ciprofloxacin, in line with ≥90% removal reported in other studies [37,38]. As suggested in the literature [39,40], conventional WWTPs likely remove fluoroquinolones primarily via sorption onto activated sludge, with biodegradation playing a secondary role. In our dataset, this interpretation is supported by the highest ciprofloxacin removal (and comparatively high removal of the other antimicrobials tested) observed at SW-WWTP, which operates a sequencing batch reactor with the highest sludge concentration and a sludge age > 60 days.

A comparatively consistent apparent removal efficiency, ranging from 64% to 85%, was observed for sulfamethoxazole, with residual concentrations in treated wastewater reaching up to 297 ng L^−1^. In contrast, no clear removal trends were recorded for macrolides, which is consistent with previous findings [41,42]. In this study, clarithromycin removal ranged from 4% to 91%. Even greater variability was observed for azithromycin, with removal efficiencies during the winter season ranging from 73% to 90%, whereas in the summer season, treatment performance fluctuated widely, with apparent removal values ranging from −289% to 61%. For erythromycin, negative removal was also noted during the winter season (−554% to −7%), while in the summer season, slightly improved removal efficiencies were observed (−40% to 39%).

Higher concentrations of macrolides in effluent than in influent indicate an apparent increase in pharmaceutical concentrations during wastewater treatment, potentially resulting from the deconjugation of metabolites and from the recirculation of reject water following sludge treatment processes such as dewatering and digestion/fermentation. This phenomenon, together with possible sorption–desorption of macrolides onto activated sludge biomass, warrants further investigation, especially because sorption via cation-exchange mechanisms has been reported [43], but is generally considered to play only a minor role in conventional WWTPs [44,45]. In this study, azithromycin concentrations in treated wastewater ranged from 769.7 to 3988.7 ng L^−1^, while those of clarithromycin ranged from 155.9 to 2866.0 ng L^−1^, values that fall within the upper range of macrolide concentrations reported in treated municipal effluents in previous European studies [8,46].

### 3.4. Concentration of Antibiotics in WWTP Receiving Waters and the Vistula Estuary

In receiving waters downstream of the studied WWTPs and in the Vistula Estuary, concentrations of the target antimicrobials were generally much lower than in treated effluents. Among the studied compounds, ciprofloxacin was not detected in the receiving waters of the tested WWTPs; however, it was found in the Vistula Estuary during the summer sampling campaign at a concentration of 0.2 ng L^−1^ (Figure 3). Overall, the highest concentrations of antimicrobial agents in the coastal receiving waters were noted in the summer season (August 2017), especially in surface water collected above the marine outflow of the largest WWTP (GW-WWTP) but did not exceed 10 ng L^−1^. During the winter season (February 2018), most target compounds were below the detection limit, with the exception of sulfamethoxazole (0.9–1.9 ng L^−1^) and azithromycin (1.1 ng L^−1^). These levels are at the lower end of those reported for European surface waters, where sulfonamides and macrolides are frequently detected in the low- to mid-ng L^−1^ range and can occasionally reach tens to hundreds of ng L^−1^ in rivers and coastal zones influenced by municipal effluents [47,48].

In the Czarna Wda River, concentrations of antimicrobials were higher downstream than upstream of the JG-WWTP discharge point, particularly for clarithromycin (16 ng L^−1^ downstream vs. up to 3.6 ng L^−1^ upstream) and sulfamethoxazole (12 ng L^−1^ downstream vs. up to 2.1 ng L^−1^ upstream). This upstream–downstream pattern is consistent with a treated-wastewater-related signal in this small river and may reflect limited dilution capacity under the sampled conditions (Figure 3). In the Vistula Estuary, all studied antimicrobials were detected during the summer campaign, with the highest concentration recorded for sulfamethoxazole (6.4 ng L^−1^). In addition to dilution and dispersion, the fate of antibiotics in receiving waters may also be influenced by processes such as sorption to and deposition in sediments, biodegradation and bioaccumulation; therefore, the environmental burden associated with these compounds warrants further detailed investigation.

### 3.5. Antibiotics Consumption Patterns in the Pomeranian Voivodeship

Given that pharmaceutical loads entering WWTPs primarily originate from community use, outpatient antibiotic consumption in the Pomeranian Voivodeship was analysed to contextualise the measured influent concentrations (Section 3.3) and to provide input data for the calculation of Predicted Incoming Loads (*PIL*s) (Section 3.6). Outpatient consumption was calculated using monthly reimbursement data obtained from the National Health Fund (NFZ) under a special request (Section 2.6).

**Figure 4:**
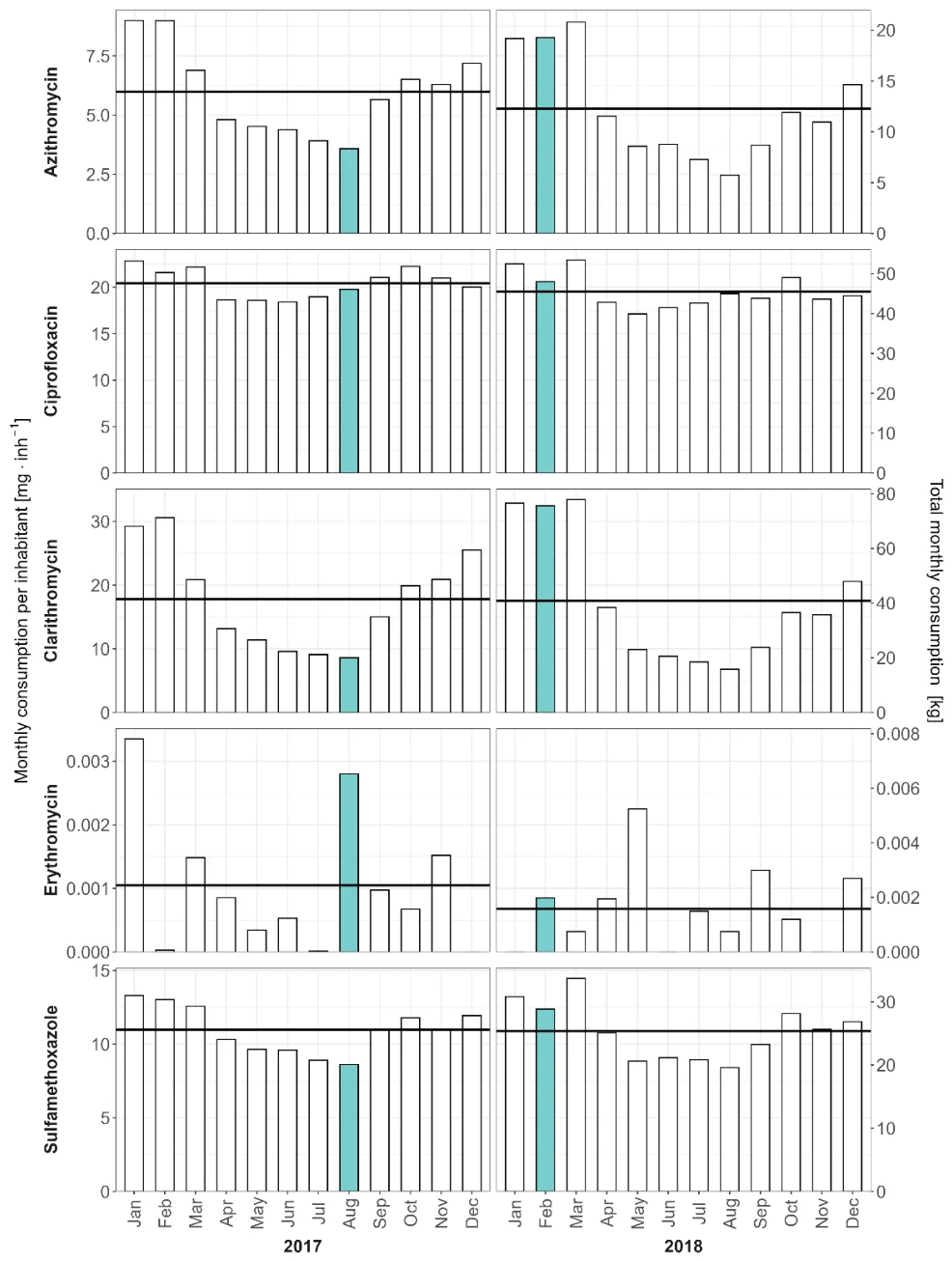
Total monthly (and per capita) outpatient consumption of macrolides (erythromycin, azithromycin, clarithromycin), ciprofloxacin, and sulfamethoxazole in the Pomeranian Voivodeship, 2017–2018; values for August 2017 and February 2018 are highlighted in green, as they were used to calculate the monthly pharmaceutical load entering the wastewater systems. The solid horizontal line denotes the average value.

Monthly per-capita outpatient use showed a clear and consistent seasonal pattern across all investigated antimicrobials in the Pomeranian Voivodeship in 2017–2018 (Figure 4). For the macrolides, winter months (January–March) exceeded the annual mean, whereas summer (June–August) fell below it, indicating a winter-dominant prescribing signal likely linked to respiratory infections. Seasonality was least pronounced for ciprofloxacin, reflecting its predominant use in urinary rather than respiratory tract infections [49]. Consistently, class-level analyses report the smallest seasonal amplitude for quinolones (≈0.081) compared with macrolides (≈0.74) and penicillins (≈1.1) [50]. Overall, total consumption in the Pomeranian Region was similar in 2017 and 2018, with the highest totals for ciprofloxacin (571.0 and 547.6 kg, respectively), followed by clarithromycin (497.8 and 491.3 kg), sulfamethoxazole (304.9 and 306.4 kg), and azithromycin (166.9 and 147.6 kg), while erythromycin did not exceed 0.02 kg in either year.

### 3.6. Calculations of Predicted Incoming Load (PIL) and Measured Incoming Load (MIL) for the Studied WWTPs

**Figure 5:**
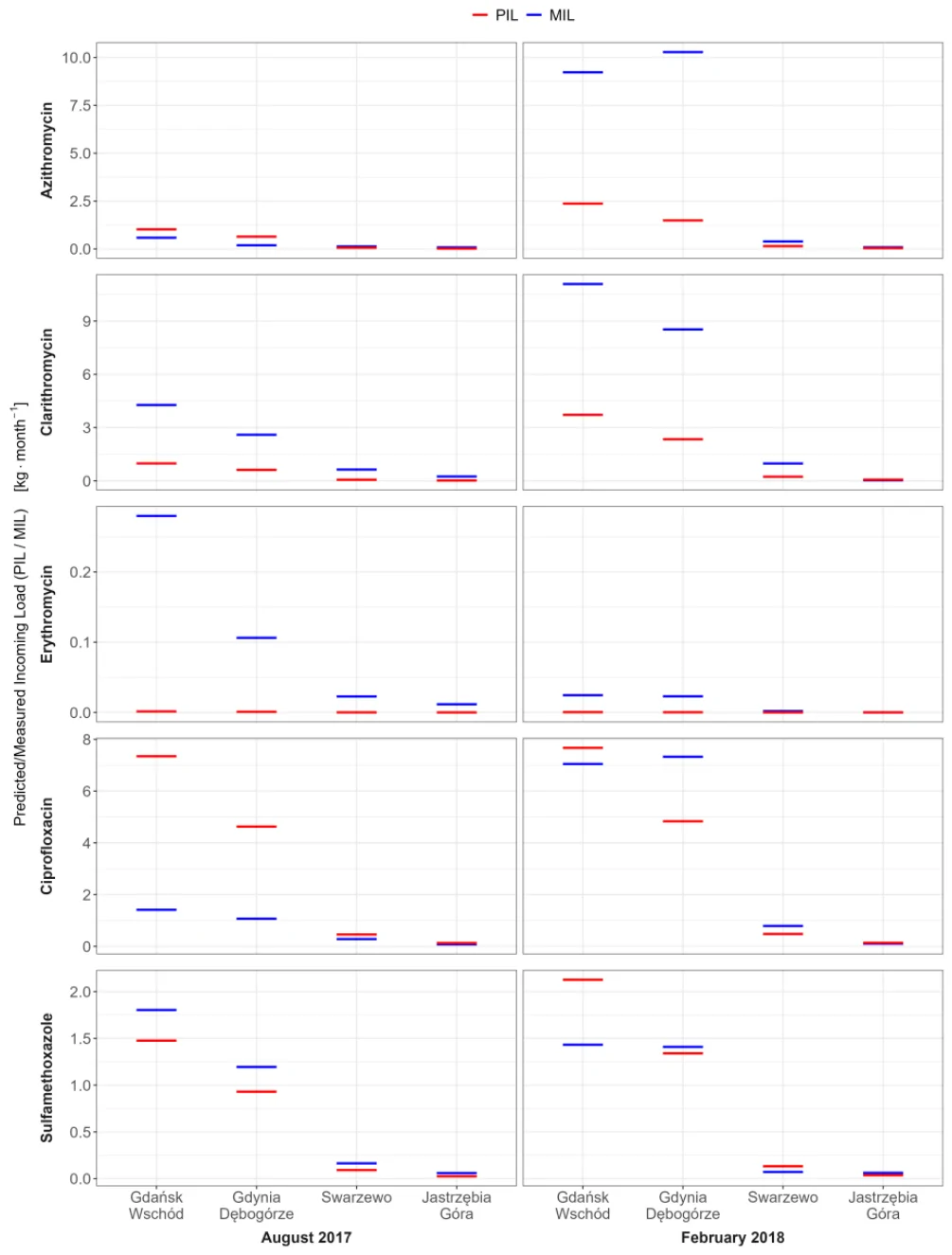
Comparison of predicted incoming loads (*PIL*s) and measured incoming loads (*MIL*s) comparison for tested WWTPs (in Gdańsk Wschód, Gdynia Dębogórze, Swarzewo and Jastrzębia Góra) calculated for data obtained in August 2017 and February 2018.

The occurrence of pharmaceuticals in urban WWTPs is expected to reflect population-level consumption patterns considering the number of inhabitants connected to the sewerage system. Thus, in the present work, the monthly outpatient consumption data for August 2017 and February 2018 (Section 3.5) were subsequently used as a cost-efficient proxy for population-level exposure to derive Predicted Incoming Loads (*PIL*s) for each WWTP catchment and to compare them with Measured Incoming Loads (*MIL*s) obtained from measured influent concentrations (Section 3.3). *PIL*s and *MIL*s were calculated for these two months using Equations (1) and (2) (see Section 2.7). The resulting values are presented in Figure 5.

Across tested antimicrobials, WWTP in Gdańsk (GW-WWTP) and in Gdynia (GD-WWTP) show a consistent pattern of *MIL* > *PIL* in both August 2017 and February 2018. This outcome is expected because *PIL*s were derived solely from reimbursed outpatient prescriptions for the general population in Pomeranian Voivodeship, whereas both catchments include multiple hospitals whose inpatient antibiotic use contributes directly to the sewer network and therefore inflates *MIL* relative to *PIL*. By contrast, for the smaller systems SW-WWTP (Swarzewo) and JG-WWTP (Jastrzębia Góra), *PIL*s and *MIL*s were broadly concordant, which is likely due to the negligible inpatient load in these catchments. In such settings, outpatient consumption represents a reasonable proxy for incoming mass loads.

Thus, for certain catchments, a consumption-based approach offers a practical means to approximate influent antimicrobial masses (*PIL*). When combined with plant-specific removal rates, *PIL* can be translated into estimated effluent loads and the resulting pressure on receiving waters. Linking outpatient use to predicted loads provides scientifically defensible preliminary estimates for first-tier screening, while accounting for key parameters affecting load estimation, including the population served, outpatient prescribing patterns, compound-specific excretion and apparent aqueous-phase removal. This rationale aligns with literature advocating consumption- or DDD-based emission modelling as a resource-efficient complement to direct measurements, supporting the prioritisation and better targeting of monitoring efforts rather than replacing detailed analytical assessment [24,51].

### 3.7. Environmental (ERA) and Antimicrobial-Resistance Risk Assessment (AMR-RA)

**Figure 6:**
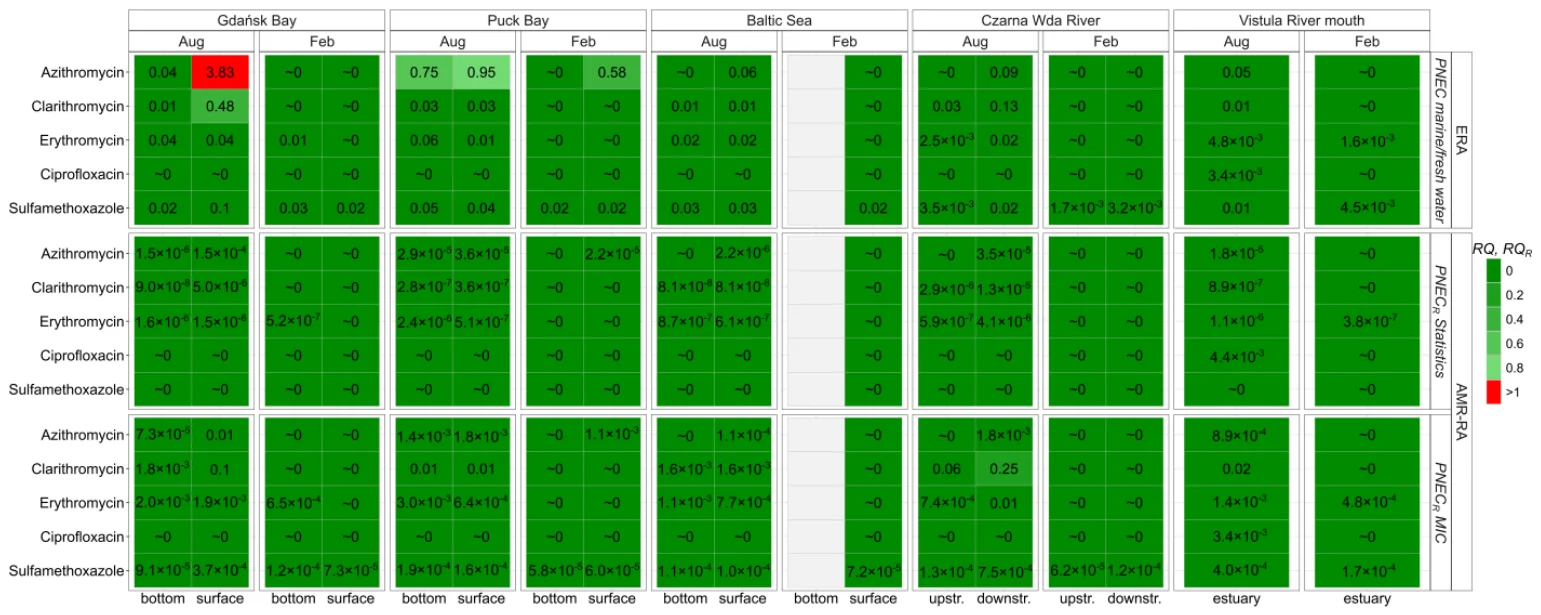
Screening-level ERA and AMR-RA risk quotients (*RQ*/*RQ*_R_) calculated for antimicrobial concentrations detected in receiving waters (*MEC*) affected by treated wastewater discharges in August 2017 and February 2018; numbers in the cells display calculated *RQ*/*RQ*_R_ values; red colour indicates *RQ* > 1, whereas ~0 denotes values close to zero. Details for *RQ*/*RQ*_R_ calculations are provided in Table 1 and Equations (3) and (4).

In this study, ERA and AMR-RA, interpreted as first-tier screening assessments, were conducted for antimicrobials detected in receiving waters impacted by WWTP effluents. Measured environmental concentrations (*MEC*s) of erythromycin, azithromycin, clarithromycin, ciprofloxacin, and sulfamethoxazole downstream of JG-WWTP and at the marine outfalls of GW-WWTP, GD-WWTP, and SW-WWTP were compared with their effect thresholds, here: *PNEC* (ecotoxicological) and *PNEC*_R_ (AMR-related), as compiled in Table 1. The MIC-derived *PNEC*_R_ approach was developed by Bengtsson-Palme and Larsson [25], while the statistic-derived *PNEC*_R_ approach was proposed by Murray et al. [26]. *MEC*s together with *PNEC* and *PNEC*_R_ values were then used to calculate *RQ* and *RQ*_R_ according to Equations (3) and (4). These calculated indicators determined for the collected samples are displayed in Figure 6. Samples were collected near the sites in which WWTP effluent is released to the receiving waters (Figure 1). At each site, two samples were collected. For marine sites, one near-bottom and one subsurface sample were collected; for the river site, samples were collected upstream and downstream of the WWTP. In addition, a sample was collected at the Vistula River mouth. For *RQ* calculations, freshwater *PNEC* values were considered the most appropriate for this site because it is a low-salinity transitional zone. This is reflected in the column headers and x-axis categories of the Figure 6. The row headers indicate the ERA and AMR-RA estimation method, whereas the y-axis categories reflect the antibiotic agent for which the value was determined. The obtained *RQ* and *RQ*_R_ values are given in the chart cells. The ERA threshold exceedance (*RQ* > 1) occurred only once, in summer (August 2017) for azithromycin in Gdańsk Bay, receiving effluent from GW-WWTP. Elevated *RQ* values for this compound were also observed in Puck Bay (recipient of GD-WWTP), reaching 0.95 in August 2017 and 0.58 in February 2018. Except for these azithromycin results, the ERA values from the remaining compounds and sites were below the low-concern threshold (*RQ* < 0.1). Similarly, for AMR-RA screening, most *RQ*_R_ values remained below low-concern threshold (*RQ*_R_ < 0.1).

It should be emphasized that *PNEC* values for marine species are generally lower than those for freshwater organisms, reflecting the high sensitivity of certain marine taxa (see Table 1). Thus, although submarine outfalls substantially reduce antimicrobial concentrations in coastal waters, even low ng L^−^^1^ concentrations of antimicrobials may approach or exceed risk thresholds in semi-enclosed marine systems such as the Baltic coastal zone, indicating that these environments remain particularly vulnerable despite significant dilution. The large differences between MIC-derived and statistic-derived *PNEC*_R_ thresholds, particularly for macrolides, highlight methodological uncertainty in current AMR-RA. At the same time, an Environment Agency review (2024) supports the use of MIC-derived *PNEC*_R_ values as a conservative first-tier benchmark, while emphasising that no fully harmonised method for deriving environmental AMR-selection thresholds has yet been established.

These *RQ*/*RQ*_R_ values should be interpreted as site- and time-specific first-tier screening and prioritisation indicators that help identify potential areas of concern and guide further assessment, rather than as standalone evidence sufficient to demonstrate ecological effects, chronic exposure-related impacts, or confirmed resistance selection. A more definitive assessment would require dedicated hydrodynamic measurements, repeated sampling and dispersion modelling.

## 4. Conclusions

This study shows that, in the context of the recast Urban Wastewater Treatment Directive (UWWTD), attention must remain focused not only on micropollutants but also on the classical objective of protecting receiving waters from eutrophication. Although all four WWTPs achieved >90% nutrient removal and complied with current discharge requirements, SW-WWTP and JG-WWTP would not meet the new stricter TN and TP thresholds, whereas GD-WWTP would fall short only of the corresponding TP threshold. These observations indicate that further optimisation of nutrient removal may be required to support future compliance, potentially in parallel with, or even before, the implementation of quaternary treatment for micropollutant removal.

For the studied antimicrobials, treatment performance in activated sludge systems was clearly compound-specific. Ciprofloxacin was removed very efficiently (>95%), sulfamethoxazole only moderately, whereas macrolides showed highly variable and sometimes even apparent negative removal, resulting in effluent concentrations in the µg L^−^^1^ range despite comparable influent levels. This pattern needs detailed investigation of internal plant processes, including metabolite deconjugation, sorption to activated sludge and recirculation of sludge side-streams. In receiving waters, low-ng L^−^^1^ concentrations were detected at offshore coastal stations, consistent with substantial dilution and dispersion by submarine outfalls under the sampled conditions, while the Czarna Wda River showed a clear wastewater signal. The presence of all target antibiotics in the Vistula Estuary also raises questions about cumulative exposure in this transitional environment.

This is particularly important given that environmental risk quotients for azithromycin approached or exceeded unity in Gdańsk and Puck Bay. That this occurred in coastal areas rather than in the river highlights a key regulatory nuance: marine *PNEC*s are often lower than fresh water benchmarks, leaving semi-enclosed coastal systems vulnerable despite favourable dilution. The microbiological data add a One Health dimension. Cefotaxime-resistant *E. coli* and faecal coliforms were consistently detected in treated wastewater and receiving waters, in some cases reaching non-negligible proportions of the faecal indicator community. Together, chemical and microbiological indicators, interpreted in their hydrological context, underscore both the need and the potential for genuinely risk-based implementation of the recast UWWTD.

In this context, a further practical insight concerns predicted incoming loads (*PIL*s), derived from outpatient antibiotic consumption, which showed closed agreement with measured incoming loads (*MIL*s) in WWTP catchments without substantial hospital contributions. In community-dominated catchments, routinely collected consumption data can act as a cost-efficient proxy for influent pharmaceutical loads and support screening-level risk assessments and the prioritisation of advanced treatment where direct monitoring is limited.

The 2017–2018 dataset should be interpreted as a retrospective pre-recast baseline rather than as a representation of current loads or current UWWTD compliance. Contemporary monitoring would be required before regulatory, permitting or investment decisions are made.

## Figures and Tables

**Table 1:** Predicted no-effect concentrations (*PNEC*s) for freshwater and marine waters, and predicted no-effect concentrations for resistance selection (*PNEC*_R_) for the antimicrobial agents evaluated in this study.

Chemical Class	Macrolides			Quinolone	Sulfonamide
Compound	Azithromycin	Clarithromycin	Erythromycin	Ciprofloxacin	Sulfamethoxazole
CAS No.	83905-01-5	81103-11-9	114-07-8	85721-33-1	723-46-6
*PNEC* (Predicted No-Effect Concentration) the lowest value for fresh and marine waters					
*PNEC* freshwater (µg L^−1^) *	0.019	0.12	0.3	0.064	0.6
*PNEC* marine water (µg L^−1^) *	0.0019	0.013	0.05	0.0064	0.06
*PNEC*_R_ (Predicted No-Effect Concentration for resistance) protective against resistance promotion					
PNEC_R_ MIC ** (µg L^−1^)	0.25	0.25	1	0.064	16
PNEC_R_ Statistics *** (µg L^−1^)	50	50	1250	0.05	-

* according to the NORMAN ecotoxicology database [18]. ** according to Bengtsson-Palme & Larsson [25]—extrapolation of MICs dataset for individual clinical strains. *** according to Murray et al. [26]—wastewater influent growth (OD) SELECT statistic experimental system.

## Data Availability

The data supporting the findings of this study are available in the Zenodo Repository at https://doi.org/10.5281/zenodo.22828829, accessed on 12 July 2026.

## Supplementary Materials

The following supporting information can be downloaded at: https://www.mdpi.com/article/10.3390/antibiotics15100963/s1, Table S1: Community and hospital antibiotic consumption in the EU/EEA and Poland, 2015–2024*, expressed in DDD per 1000 inhabitants per day (DID), and percentage (%) of WHO AWaRe Reserve group antimicrobials consumed out of total hospital sector AWaRe consumption; Table S2a: Characteristics of the studied WWTPs; Table S2b: Discharge limits used for comparison in this study and the corresponding values presented for the recast UWWTD (EU) 2024/3019), which Member States must transpose its main provisions into national law by 31 July 2027, changes highlighted; Table S2c: Physical and chemical characterization of sampling points during summer campaign August 2017; Table S2d: Physical and chemical characterization of sampling points during winter campaign February 2018; Table S2e: Estimated monthly loads of basic parameters discharged from the studied WWTPs into receiving waters in August 2017 and February 2018; Table S3: Total (urine + faeces) unchanged excretion rate reported in the literature and used in this study to calculate predicted incoming loads (*PILs*) for selected antimicrobials; Figure S1: Basic parameters in inlet and outlet of WWTPs in: Gdańsk (Gdańsk Wschód WWTP), Gdynia (Gdynia Dębogórze WWTP), Swarzewo (Swarzewo WWTP) and Jastrzębia Góra (Jastrzebia Góra WWTP), as well as in their receiving waters; surface and bottom samples of marine outfalls in Gdańsk Bay, Puck Bay, and Baltic Sea; downstream and upstream the discharge point at Czarna Wda River; additionally, the results are presented for the Vistula River mouth (see Figure 1 for details); Comments. Refs [52,53,54,55,56,57] are cited in Supplementary Materials file.

## Abbreviations

The following abbreviations are used in this manuscript:

AF	Assessment factor
AMR	Antimicrobial resistance
AMR-RA	Antimicrobial-resistance risk assessment
ATC	Anatomical Therapeutic Chemical classification system
AWaRe	Access, Watch and Reserve classification
BOD_5_	Five-day biochemical oxygen demand
BOT	Bottom-water sample
CAS	Chemical Abstracts Service
CFU	Colony-forming unit
COD	Chemical oxygen demand
CTX-R	Cefotaxime-resistant
CW	Czarna Wda River
CW-DOWN	Sampling point downstream of the wastewater discharge in the Czarna Wda River
CW-UP	Sampling point upstream of the wastewater discharge in the Czarna Wda River
DDD	Defined daily dose
DID	Defined daily doses per 1000 inhabitants per day
EAN	European Article Number
ECDC	European Centre for Disease Prevention and Control
EDTA	Ethylenediaminetetraacetic acid
EEA	European Economic Area
EEC	European Economic Community
EMA	European Medicines Agency
ERA	Environmental risk assessment
ESBL	Extended-spectrum β-lactamase
EU	European Union
EUCAST	European Committee on Antimicrobial Susceptibility Testing
FAO	Food and Agriculture Organization of the United Nations
FC	Faecal coliforms
GD-WWTP	Gdynia-Dębogórze Wastewater Treatment Plant
GLASS	Global Antimicrobial Resistance and Use Surveillance System
GW-WWTP	Gdańsk-Wschód Wastewater Treatment Plant
HPLC	High-performance liquid chromatography
IC	Ion chromatography
IS	Internal standard
ISO	International Organization for Standardization
JG-WWTP	Jastrzębia Góra Wastewater Treatment Plant
*MEC*	Measured environmental concentration
m-FC	Membrane faecal coliform agar
MIC	Minimum inhibitory concentration
*MIL*	Measured incoming load
MQL	Method quantification limit
MSS	Mineral suspended solids
NFZ	National Health Fund (Narodowy Fundusz Zdrowia)
NOEC	No-observed-effect concentration
NOEC_R_	No-observed-effect concentration for resistance selection
NORMAN	NORMAN Network—European network and database for emerging environmental substances
PE	Population equivalent
PEC	Predicted environmental concentration
*PIL*	Predicted incoming load
*PNEC*	Predicted no-effect concentration
*PNEC*_R_	Predicted no-effect concentration for resistance selection
*RQ*	Risk quotient
*RQ*_R_	Risk quotient for resistance selection
SELECT	SELection End points in Communities of bacTeria
SPE	Solid-phase extraction
SRM	Standard Reference Material
SUR	Surface-water sample
SW-WWTP	Swarzewo Wastewater Treatment Plant
TN	Total nitrogen
TP	Total phosphorus
TSS	Total suspended solids
UNEP	United Nations Environment Programme
UPLC-ESI-MS/MS	Ultra-high-performance liquid chromatography–electrospray ionization–tandem mass spectrometry
UWWTD	Urban Wastewater Treatment Directive
VR	Vistula River
VR-M	Vistula River mouth
VSS	Volatile suspended solids
WHO	World Health Organization
WOAH	World Organisation for Animal Health
WWTP	Wastewater treatment plant
