# Supplementary material for: From Outpatient Consumption to Receiving Waters: A Retrospective Screening Assessment of Antibiotic Loads, Resistance Indicators, and Implications for the Recast Urban Wastewater Treatment Directive
Source: Antibiotics (Basel). 2026 Sep 29;15(10):963. doi: 10.3390/antibiotics15100963 (PMC13700695; doi:10.3390/antibiotics15100963)
Supplement: Supplementary file 1 [file antibiotics-15-00963-s001.zip › antibiotics-4471372-supplementary.pdf]

Supplementary materials for:

From outpatient consumption to receiving waters: a retrospective screening assessment of antibiotic loads, resistance indicators, and implications for the recast Urban Wastewater Treatment Directive

Aneta Łuczkiewicz [1, *], Wojciech Artichowicz [1], Małgorzata Szopińska[1], Katarzyna Jankowska[1], Ola Svahn[2], Erland Björklund [3], Ewa Kotlarska [4, *] , Nikol Szeszuła [1], and Sylwia Fudala-Książek[1]

1 Faculty of Civil and Environmental Engineering, Gdansk University of Technology, Narutowicza 11/12, Gdansk 80-233, Poland; aneta.luczkiewicz@pg.edu.pl; wojciech.artichowicz@pg.edu.pl; katarzyna.jankowska@pg.edu.pl; malgorzata.szopinska@pg.edu.pl; s189199@student.pg.edu.pl; sylwia.fudala-ksiazek@pg.edu.pl;
2 Faculty of Natural Science, Kristianstad University, SE-291 39 Kristianstad, Sweden; ola.svahn@hkr.se;
3 Malmö University, Malmö, Sweden; erland.bjorklund@mau.se;
4 Genetics and Marine Biotechnology Department, Institute of Oceanology Polish Academy of Sciences, Powstańców Warszawy 55, 81-712 Sopot, Poland; ekotlarska@iopan.pl;
* Correspondence: AL: aneta.luczkiewicz@pg.edu.pl; EK: ekotlarska@iopan.pl

Tables

Table S1 Community and hospital antibiotic consumption in the EU/EEA and Poland, 2015–2024[*], expressed in DDD per 1,000 inhabitants per day (DID), and percentage (%) of WHO AWaRe Reserve group antimicrobials consumed out of total hospital sector AWaRe consumption

Year	EU/EEA average			Year	Poland		
	Community	Hospital	AWaRe Reserve		Community	Hospital	AWaRe Reserve
	DID	DID	%		DID	DID	%
2015	22.4	2.04	2.2	2015	26.2	1.31	0.8
2016	21.9	2.06	2.3	2016	24.0	1.25	1.1
2017	21.8	2.03	2.6	2017	27.0	1.62	0.8
2018	18.4	1.79	3.3	2018	23.0	1.36	1.6
2019	18.0	1.77	3.9	2019	22.2	1.42	1.0
2020	15.0	1.59	4.0	2020	17.1	1.36	1.3
2021	15.2	1.55	4.6	2021	19.2	1.40	1.6
2022	18.1	1.61	5.0	2022	22.7	1.33	1.9
2023	18.5	1.66	5.4	2023	21.8	1.39	2.1
2024	18.8	1.67	5.4	2024	21.2	1.42	2.3

*) according to: European Centre for Disease Prevention and Control (ECDC). *Antimicrobial consumption in the EU/EEA (ESAC-Net) – Annual Epidemiological Report 2015–2024*. Stockholm: ECDC; 2025. Available from: https://www.ecdc.europa.eu/en/publications-data/monitoring/all-annual-epidemiological-reports [accessed 12 July 2026].

Table S2a. Characteristics of the studied WWTPs

Parameter	WWTP - wastewater treatment plant			
	Jastrzębia Góra JG-WWTP	Swarzewo SW-WWTP	Gdynia–Dębogórze GD-WWTP	Gdańsk-Wschód GW-WWTP

Population connected to the sewerage system	~ 10, 000	~ 35, 668	~ 360, 000	~ 571, 350
Designed flow and population-equivalent capacity	7, 305 m^3/d<br>62, 000 PE	18, 000 m^3/d<br>180, 000 PE	73, 000 m^3/d<br>440, 000 PE	120, 000 m^3/d<br>840, 200 PE
Average flow in sampled months: August 2017/ February 2018	$Q_{Aug.}$= 5 010 m^3 d^{-1}<br>$Q_{Febr.}$= 1 108 m^3 d^{-1}	$Q_{Aug.}$= 8 560 m^3 d^{-1}<br>$Q_{Febr.}$= 4 788 m^3 d^{-1}	$Q_{Aug.}$= 61 233 m^3 d^{-1}<br>$Q_{Febr.}$= 60, 823 m^3 d^{-1}	$Q_{Aug.}$= 95 958 m^3 d^{-1}<br>$Q_{Febr.}$= 88, 771 m^3 d^{-1}
Hospital/industrial wastewater	none	< 1%/5%<br>food & cosmetics pharmaceutical, industry	0.1%/10%<br>shipyards, food & chemical, cosmetics industry	1%/11%<br>shipyards, food & chemical industry
Septic tanks (% of total flow)	~ 4.7% of total flow	~ 3.5% of total flow	~ 1% of total flow	~ 0.14% of total flow
Mechanical treatment	screens with combined grit trap and grease separation	Coarse debris screen; Chamber for sand and grit removal;	hook screens, grit chamber with a grease trap, radial primary settling tanks.	mechanical screens, aerated grit chamber with a grease trap, radial primary settling tanks
Biological treatment	5-stage (modified) Bardenpho process (pre-denitrification, dephosphatation, denitrification and nitrification and radial secondary settling tanks	6 SBR (sludge age about 63 days); nitrification-denitrification process; methanol used as external carbon source	Bardenpho system with simultaneous denitrification in Caroussel system (advanced nutrients removal) secondary settling tanks (sludge age about 29 days)	biological reactors working in an anaerobic/anoxic/ oxic (A2/O) system (advanced biological nutrients removal), radial secondary settling tanks
Additional/ chemical treatment	UV disinfection prior to discharge to the receiver.	precipitation of phosphorus supported with PIX	PIX dosing system for occasional phosphorus removal	PIX dosing system for occasional phosphorus removal
primary and excessive sludge treatment	aerobic stabilization with lime (CaO hygienisation)	co-digested with organic waste in an mesophilic regime (38÷40 °C)	digestion in the mesophilic regime (32–38 °C) and incineration	digestion in the mesophilic regime (38–40 °C) and incineration
Recipient	Czarna Wda River – via point discharge (CW)	coastal waters of Baltic Sea by 1.4 km submarine collector (BS)	coastal waters of Puck Bay by 2.3 km submarine collector (PB)	coastal waters of Gdansk Bay by 2.3 km submarine collector (GB)

Table S2b. Discharge limits used for comparison in this study and the corresponding values presented for the recast UWWTD (EU) 2024/3019), which Member States must transpose its main provisions into national law by 31 July 2027; changes highlighted

WWTP	Czarna Wda River – via point discharge (CW)	coastal waters of Baltic Sea by 1.4 km submarine collector (BS)	coastal waters of Puck Bay by 2.3 km submarine collector (PB)	coastal waters of Gdansk Bay by 2.3 km submarine collector (GB)
Load capacity	62, 000 PE	180, 000 PE	440, 000 PE	840, 200 PE
TSS	35 mg/L (≥90% removal)			
BOD_5	15 mg O_2/L (≥90% removal) / **25 mg O_2/L (70%-90% removal)**			
COD	125 mg O_2/L (≥75% removal) / **TOC 37 mg/L** or **≥75%** allowed as explicit alternative			
TN	15 mg N/L (70%-80% removal) / **10**	10 [mg N /L] (70%-80% removal) / **8 mg/L** (**≥80%** removal)		

	**mg/L and ≥80% removal**	
TP	2 mg P/L (80% removal) / **0.7 mg/L (≥87.5% removal)**	1 [mg/L] (80% removal) / **0.5 mg/L** (**≥90%** removal)

Table S2c Physical and chemical characterization of sampling points during summer campaign August 2017

Sampling place		date	pH	Conduct [mS/cm]	redox [mV]	$P-PO_4$ [mg/L]	$N-NH_4$ [mg/L]	$N-NO_3$ [mg/L]	$N-NO_2$ [mg/L]	COD [mg/L]	BOD_5 [mg/L]	SO_4^{2-} [mg/L]	Cl^- [mg/L]	TSS [mg/L]
GW-WWTP	Inflow (24h-flow-proportional)	28/29.08	7.7	1.48	10	4.59	59.4	0.987	0.025	765	360	ND	ND	340
	Outflow (24h-flow-proportional)	28/29.08	7.9	0.975	ND	0.05	0.15	3.98	0.043	27.8	2.2	69.6	112	4.4
	Marine outflow - surface water	30.08	8.1	10.94	44.8	0.038	0.035	0.011	0.004	22.4	2	564	4325	1.4
	Marine outflow - bottom water	30.08	8.2	11.42	47.7	0.036	0.037	0.029	0.003	51.1	2.2	580	3687	2.6
GD-WWTP	Inflow (24h-flow-proportional)	28/29.08	7.5	1.39	12	4.6	57.2	1.3	0.26	1200	430	ND	ND	540.0
	Outflow (24h-flow-proportional)	28/29.08	8.0	0.916	ND	0.95	0.42	4.8	0.11	34.9	3	105	111	5.0
	Marine outflow - surface water	30.08	8.4	10.93	154.1	0.007	0.035	0.113	0.008	28.4	3.6	526	3758	3
	Marine outflow - bottom water	30.08	8.4	10.32	159.4	0.011	0.032	0.087	0.009	12.4	1.2	555	4325	16
SW-WWTP	Inflow (24h-flow-proportional)	23/24.08	7.1	2.35	-307.9	6.67	83.8	1.64	0.545	1375	800	239	404	390.0
	Outflow (24h-flow-proportional)	24/25.08	7.6	1.376	22.9	0.370	0.285	9.95	0.152	29.9	3.4	109	284	1.3
	Marine outflow - surface water	25.08	8.0	11.04	28	0.016	0.019	0.093	0.009	34	3.6	129	4254	2.2
	Marine outflow - bottom water	25.08	7.9	11.34	39.4	0.017	0.019	0.075	0.008	33	2.1	490	4254	3.8
JG-WWTP	Inflow (24h-flow-proportional)	27/28.08	7.5	1.388	-298.5	6.96	63.4	0.781	0.297	1184	945	192	113.4	343.3
	Outflow (24h-flow-proportional)	28/29.08	7.4	0.894	29.4	0.585	0.068	3.84	0.03	31.6	3.9	76.5	141.8	3.3
	Upstream 0.1 m below water level*	29.08	7.6	1.024	45.7	0.163	0.044	0.523	0.028	22.5	2.5	39.4	14.2	2.7
	Downstream 0.1 m below water level*	29.08	7.7	1.061	34.4	0.216	0.047	0.881	0.026	24.9	3.1	40.5	14.2	3.5
VR	Estuary - surface water	30.08	8.3	8.00	45.4	0.11	0.043	0.023	0.004	32.8	3.6	419	2198	2.6

ND – not determined <DL – below detection limit

Table S2d Physical and chemical characterization of sampling points during winter campaign February 2018

Sampling place		date	pH	Conduct. [mS/cm]	redox [mV]	$P\text{-}PO_4$ [mg/L]	$N\text{-}NH_4$ [mg/L]	$N\text{-}NO_3$ [mg/L]	$N\text{-}NO_2$ [mg/L]	COD [mg/L]	BOD_5 [mg/L]	SO_4^{2-} [mg/L]	Cl^- [mg/L]	TSS [mg/L]
GW-WWTP	Inflow (24h-flow-proportional)	27/28.02	7.8	1.680	21	5.4	65.2	ND	ND	1000	721	ND	ND	470
	Outflow (24h-flow-proportional)	27/28.02	7.8	1.250	ND	0.05	<0.015	5.36	0.21	36.5	4.1	94.3	161	6.7
	Marine outflow - surface water	28.02	8.1	10.00	115.9	0.07	0.027	0.093	<0.015	0.393	< DL	3878	586	2.5
	Marine outflow - bottom water	28.02	7.1	13.21	117.8	0.08	0.036	0.056	<0.015	1.16	0.69	4428	677	2.5
GD-WWTP	Inflow (24h-flow-proportional)	27/28.02	ND	ND	15	5.43	66.7	ND	ND	1000	470	ND	ND	470
	Outflow (24h-flow-proportional)	27/28.02	7.9	1.151	ND	0.67	**0.33**	3.80	0.0720	36.9	3.2	147	ND	<5
	Marine outflow - surface water	28.02	7.3	12.35	123.5	0.09	<0.015	0.047	<0.015	0.302	< DL	4104	622	1.7
	Marine outflow - bottom water	28.02	8.0	12.27	95.5	0.06	0.023	0.047	<0.015	0.324	< DL	1213	317	54.9
SW-WWTP	Inflow (24h-flow-proportional)	27/28.02	7.8	3.08	-34.4	10.86	92	2.37	0.184	920	644	545	128	624
	Outflow (24h-flow-proportional)	27/28.02	7.7	2.37	56.9	9.53	0.638	10.5	0.081	20.01	13.8	456	245	12.7
	Marine outflow - surface water	28.02	7.4	12.24	123.8	0.08	0.296	0.198	<0.015	0.322	< DL	3886	585	6
	Marine outflow - bottom water	28.02	The samples were not collected due to adverse weather conditions.											
JG-WWTP	Inflow (24h-flow-proportional)	27/28.02	7.3	1.255	-64.8	7.08	58.5	0.851	0.177	982	687.4	111	137	330
	Outflow (24h-flow-proportional)	27/28.02	7.4	0.883	116.6	7.74	0.179	3.46	0.027	26.2	16.9	136	186	29.6
	Upstream 0.1 m below water level*	28.02	7.3	0.449	142.4	0.28	0.271	1.55	<0.015	0.074	< DL	16	93	1.7
	Downstream 0.1 m below water level*	28.02	7.4	0.437	136.2	0.25	0.279	1.48	<0.015	0.075	< DL	15	94	4.7
VR	Estuary - surface water	28.02	8.1	10.76	101.3	<0.05	<0.015	<0.23	<0.015	0.404	< DL	3547	534	3.9

ND – not determined <DL – below detection limit

Table S2e. Estimated monthly loads of basic parameters and faecal indicators discharged from the studied WWTPs into receiving waters in August 2017 and February 2018.

WWTP	**Jastrzebia Gora JG-WWTP**		**Swarzewo SW-WWTP**		**Gdynia–Debogorze GD-WWTP**		**Gdansk-Wschod GW-WWTP**	
**receiving waters**	Czarna Wda River (CW)		coastal waters of Baltic Sea (BS)		coastal waters of Puck Bay (PB)		coastal waters of Gdansk Bay (GB)	
**date**	08.2017	02.2018	08.2017	02.2018	08.2017	02.2018	08.2017	02.2018
monthly loads of basic parameters [Mg/month]								
TP	0.12	0.02	0.10	0.14	1.82	1.45	0.89	0.70
TN	0.91	0.13	2.76	1.39	13.29	10.22	17.94	19.88
COD	4.91	0.81	7.93	2.68	66.24	62.84	82.70	90.72
BOD	0.61	0.52	0.90	1.85	5.69	5.45	6.54	10.19
TSS	0.51	0.92	0.34	1.70	9.49	6.81	13.09	16.65
monthly loads of faecal indicators [CFU/month]								
FC	1.7E+14	3.9E+12	5.7E+14	3.2E+13	5.8E+15	9.0E+15	1.3E+16	4.2E+15
E. coli	2.6E+13	2.1E+12	4.0E+14	2.0E+13	3.0E+15	4.7E+15	8.4E+15	2.4E+15
CTX-R FC	3.5E+13	9.3E+10	7.1E+13	9.1E+12	5.3E+14	2.2E+15	3.9E+15	2.4E+15
CTX-R *E. coli*	1.8E+12	4.1E+10	2.0E+13	7.1E+12	2.9E+14	1.3E+15	1.3E+15	7.2E+14

Table S3 Total (urine + feces) unchanged excretion rate reported in the literature and used in this study to calculate predicted incoming loads (*PILs*) for selected antimicrobials

Drug	Unchanged (%)		Used in the study (%)	Notes	References
	urine	Feces			
**Macrolides**					
Azithromycin	~6	~50%	50%	Urine fraction small; majority via bile/feces	[1, 6]
Clarithromycin	~18 %	~4%	20%	Renal route substantial; biliary important but small	[2, 6]
Erythromycin	5–15%	~80%	90%	Small urinary fraction; biliary excretion predominant	[3, 6]
**Fluoroquinolones**					
Ciprofloxacin	30-45%	~30%	65%	Well-characterized mass balance	[4, 6]
**Sulfonamides**					
Sulfamethoxazole	~30%	~ 0%	30%	Recovered in the urine as sulfamethoxazole ~30% and ~55% as N4-acetylated metabolite; faecal fraction minor	[5, 6]

This table summarizes the proportion of an administered dose of selected antimicrobial agents that is excreted unchanged (parent drug) in humans via urine and feces, based on published pharmacokinetic data. Term 'used in the study' refers to excretion rate used to calculate the predicted incoming load (PIL).

References:

1. Fischer, J.H.; Sarto, G.E.; Habibi, M.; Kilpatrick, S.J.; Tuomala, R.E.; Shier, J.M.; Wollett, L.; Fischer, P.A.; Khorana, K.S.; Rodvold, K.A. Influence of Body Weight, Ethnicity, Oral Contraceptives, and Pregnancy on the Pharmacokinetics of Azithromycin in Women of Childbearing Age. *Antimicrob Agents Chemother* **2012**, *56*, 715-724.
2. Davey P.G. The pharmacokinetics of clarithromycin and its 14-OH metabolite. *J Hosp Infect* **1991** *19* Suppl A, 29-37.
3. Farzam, K., Jan, A. Erythromycin. In: *StatPearls* [Internet]. Treasure Island (FL): StatPearls Publishing. Available at: https://www.ncbi.nlm.nih.gov/books/NBK532249/ [Accessed 12 July 2026].
4. Gonzalez, M.A.; Uribe, F.; Moisen, S.D.; Fuster, A.P.; Selen, A.; Welling, P.G.; Painter, B. Multiple-dose pharmacokinetics and safety of ciprofloxacin in normal volunteers. *Antimicrob Agents Chemother* **1984** *26*, 741-744.
5. FDA Approved Drug Products: Bactrim (sulfamethoxazole/trimethoprim) oral tablets. https://www.accessdata.fda.gov/drugsatfda_docs/label/2014/017377s074lbl.pdf [Accessed 12 July 2026].
6. DrugBank Online, 2026. https://go.drugbank.com [Accessed 12 July 2026].

Figures

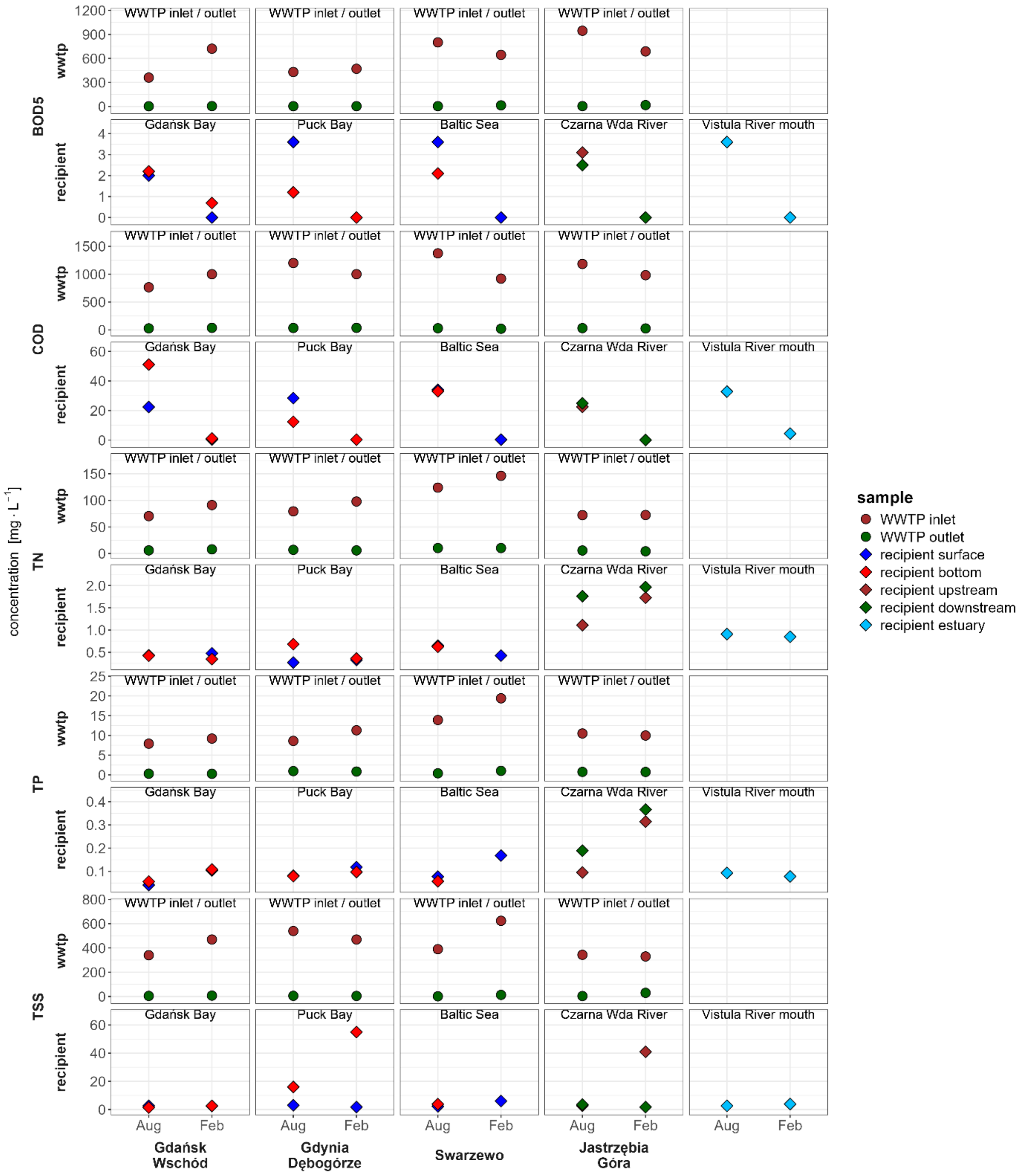

Figure S1 Basic parameters in inlet and outlet of WWTPs in: Gdańsk (Gdańsk Wschód WWTP), Gdynia (Gdynia Dębogórze WWTP), Swarzewo (Swarzewo WTP) and Jastrzębia Góra (Jastrzebia Góra WWTP), as well as in their receiving waters; surface and bottom samples of marine outfalls in Gdańsk Bay, Puck Bay, and Baltic Sea; downstream and upstream the discharge point at Czarna Wda River; additionally the results are presented for Vistula River mouth (see Figure 1 for details)

Comments

During the peak summer tourist season, both the Swarzewo WWTP (SW-WWTP) and Jastrzębia Góra WWTP (JG-WWTP) experienced hydraulic overloads, with influent volumes increasing up to tenfold, a substantial portion of which consisted of high-strength wastewater originated from septic tanks. This was evidenced by redox potential values below 0 mV, recorded in the influent streams of both plants, indicating anaerobic conditions (Table S2c-d)). No major technological issues were observed at the Gdańsk-Wschód (GW-WWTP) and Gdynia Dębogórze (GD-WWTP); however, the low BOD_5/COD ratio (< 0.5) recorded at the GD-WWTP inflow suggests a high proportion of poorly biodegradable industrial wastewater (Figure S1). When such compounds enter municipal treatment systems, they may persist through the treatment process, remain non-biodegraded, and ultimately be discharged into receiving waters, potentially leading to unforeseen environmental consequences. Notably, both facilities serve populations exceeding 150,000 PE, which, under the revised UWWTD, qualifies them for the mandatory implementation of advanced (fourth-stage) treatment targeting the removal of micropollutants.

Despite the high efficiency of nutrient removal—exceeding 90%—achieved by the studied WWTPs, significant loads of biogenic substances continue to be discharged into the receiving waters (Table S2e). As an example, in August 2017 the two largest wastewater treatment plants — GW-WWTP (840,200 PE) and GD-WWTP (440,000 PE) — discharged more than 31 Mg of total nitrogen and 2.5 Mg of total phosphorus into the Gulf of Gdańsk and its inner basin, the Puck Bay. In addition to nutrients, in August 2017 the WWTPs jointly discharge over 22 Mg of TSS, and approximately 150 Mg of organic matter, expressed as COD. Of this organic load, approximately 10% was biodegradable, as indicated by BOD measurements. Furthermore, additional nutrient inputs—approximately an order of magnitude lower—enter the Baltic Sea from smaller WWTP: the SW-WWTP and JG-WWTP, via direct discharge and indirectly through the Czarna Wda River, respectively. These cumulative discharges underscore the ongoing challenges in mitigating pollution of the Baltic Sea coastal areas.
